# Heart failure, inflammation and exercise

**DOI:** 10.7150/ijbs.109917

**Published:** 2025-04-28

**Authors:** Qian Luo, Qing Zhang, Youli Kong, Shiqi Wang, Quan Wei

**Affiliations:** 1 Rehabilitation Medicine Center and Institute of Rehabilitation Medicine, West China Hospital, Sichuan University, Chengdu 610041, China.; 2 Key Laboratory of Rehabilitation Medicine in Sichuan Province, West China Hospital, Sichuan University, Chengdu 610041, China.

**Keywords:** heart failure, inflammation, exercise, signaling pathway

## Abstract

Heart failure (HF) is a condition characterized by high morbidity, mortality, and a substantial healthcare burden, in which inflammation plays a pivotal role. This review provides a comprehensive overview of inflammation in HF progression, highlighting the dynamic alterations in immune cell populations—such as monocytes/macrophages and neutrophils—and regulatory mechanisms of key signaling pathways, including JAK and NLRP3. Furthermore, the clinical relevance of inflammatory biomarkers in predicting disease prognosis is also discussed. Emerging evidence indicates that exercise intervention can enhance cardiac function by promoting the expression of anti-inflammatory cytokines (e.g., IL-10) and mitigating myocardial fibrosis, oxidative stress, and apoptosis. Future studies should investigate how exercise modulates critical inflammatory pathways—such as TLR/MyD88/NF-κB and the NLRP3 inflammasome—and aim to establish personalized exercise protocols tailored to patients' inflammatory profiles and disease stages. Such insights may pave the way for innovative therapeutic strategies in HF management.

## 1. Introduction

Heart failure (HF) is a multifaceted clinical syndrome and represents the final common endpoint of numerous cardiovascular conditions, including coronary artery disease, hypertension, and cardiomyopathy. Affecting over 56 million individuals globally, with a prevalence of 1%-2%, HF has emerged as a significant public health challenge^1^, ^2^. In addition to its direct impact on morbidity and mortality rates, HF places a considerable socioeconomic burden due to frequent hospitalizations and rising healthcare expenditures, underscoring the pressing need for effective management strategies^3^.

Among the many pathophysiological contributors to HF, inflammation has gained prominence—not merely as a hallmark feature but as an active driver of disease progression^4^. Following myocardial injury, the innate immune response initiates cytoprotective processes aimed at restoring homeostasis^5^. However, when the inflammatory response becomes chronic or dysregulated, it leads to sustained cellular damage and adverse cardiac remodeling, thereby accelerating HF onset and deterioration^6^. This dualistic nature of inflammation—as both a reparative and pathological force—makes it an attractive therapeutic target in HF.

Exercise intervention, a non-pharmacological approach, has gained recognition for improving clinical outcomes in HF patients, including enhanced exercise tolerance, reduced rehospitalization rates, and better quality of life^7^. While those benefits are well-documented, the molecular mechanisms underlying exercise-mediated improvements—particularly its anti-inflammatory effects—remain incompletely understood. This gap underscores the importance of elucidating how exercise modulates inflammatory pathways to delay HF progression.

This article provides a systematic review of the relationship between inflammation and HF pathophysiology, focusing on molecular mechanisms linking chronic inflammation to cardiac dysfunction. This article explores exercise intervention as a regulator on inflammatory responses, detailing its potential to disrupt pro-inflammatory signaling pathways while facilitating tissue repair. Additionally, the study examines the clinical relevance of inflammatory biomarkers, evaluating their utility in assessing cardiac function and predicting HF prognosis. Finally, the article proposes future research directions aimed at advancing therapeutic strategies targeting inflammation in HF. These efforts aim to enhance patient outcomes and improve quality of life through innovative, mechanism-driven approaches.

## 2. Inflammation in the pathology of heart failure

**Figure 1:**
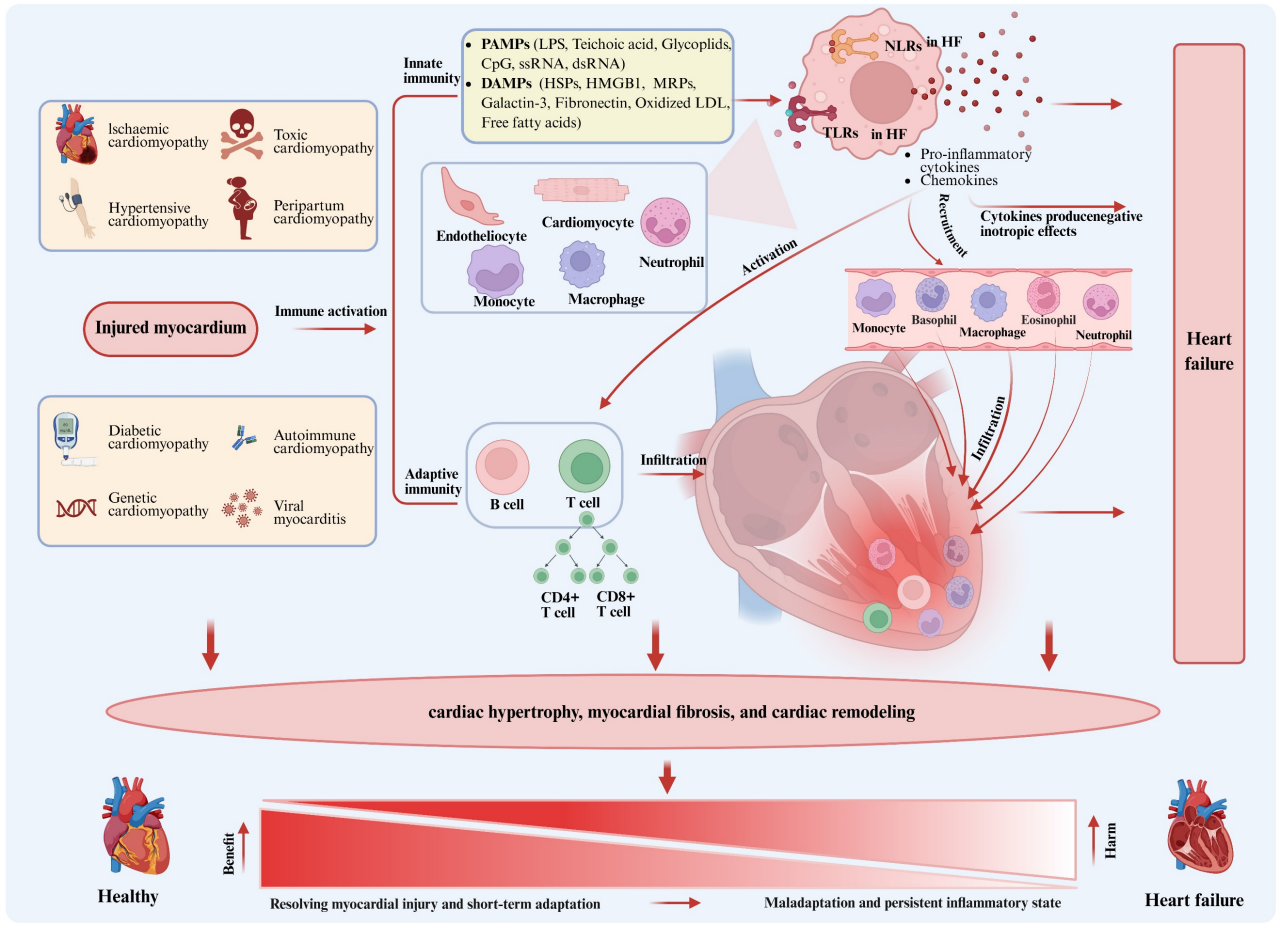
** Role of inflammation in heart failure.** Figure 1 illustrates the central role of inflammation in the pathogenesis of heart failure. Myocardial injury due to various causes activates the innate immune system, leading to the expression of damage-associated molecular patterns (DAMPs) or pathogen-associated molecular patterns (PAMPs) by cardiomyocytes, endothelial cells, and resident immune cells in response to stimuli. Those patterns are recognized by pattern recognition receptors (PRRs), such as Toll-like receptors (TLRs) and NOD-like receptors (NLRs). The activation of those receptors initiates a signaling cascade that promotes the gene expression of pro-inflammatory cytokines and chemokines, activating B cells and T cells, and promoting the recruitment of circulating neutrophils and monocytes/macrophages to the myocardium, thereby triggering adaptive immunity. The primary function of the inflammatory response is to resolve myocardial injury, allowing the heart to adapt to abnormal conditions in the short term and restore homeostasis and cardiovascular function in the long term. However, if the abnormal condition persists, a persistent inflammatory state in the tissue can have adverse effects on cardiomyocytes and the extracellular matrix, leading to progressive left ventricular remodeling and dysfunction, ultimately resulting in heart failure due to maladaptation. PAMPs, pathogen-associated molecular patterns; LPS, lipopolysaccharide; CpG, cytosine-phosphate-guanine; DAMPs, damage-associated molecular patterns; HSPs, heat-shock proteins; HMGB1, high mobility group box 1; MRPs, multidrug resistance proteins; LDL, low-density lipoprotein; NLRs, NOD-like receptors; TLRs, Toll-like receptors.

The pathogenesis of heart failure (HF) involves multiple factors, with the inflammatory response playing a central role, as shown in Figure 1. Myocardial damage due to a variety of causes activates the innate immune system, marked by the expression of damage-associated molecular patterns (DAMPs) or pathogen-associated molecular patterns (PAMPs) by cardiomyocytes, endothelial cells and resident immune cells in response to stimulating agents. These molecules are recognized by pattern recognition receptors (PRRs), such as Toll-like receptors (TLRs) and NOD-like receptors (NLRs). Activation of those receptors initiates a signaling cascade that promotes the expression of pro-inflammatory cytokines and chemokines, leading to the activation of B cells and T cells, while promoting the recruitment of circulating neutrophils and monocytes/macrophages to the myocardium to trigger adaptive immunity^5^. Activated immune cells and cytokines promote pro-hypertrophic and profibrotic signals, inducing cardiac hypertrophy and trigger cardiac fibrosis and remodeling. The main function of the inflammatory response is to resolve myocardial injury, allowing the heart to adapt to abnormal conditions in the short term and restore homeostasis and cardiovascular function in the long term. However, if the abnormal condition persists, a persistent inflammatory state within the tissue can lead to detrimental effects on cardiomyocytes and extracellular matrix, resulting in progressive left ventricular remodeling and dysfunction, and ultimately leading to HF due to maladaptation^8^. Currently, clinical studies have shown that patients with HF exhibit persistent low-grade inflammation in their hearts, which significantly contributes to the pathogenesis of HF and may lead to adverse clinical outcomes. Unfortunately, the exact timing at which this inflammatory response switches from beneficial to detrimental remains elusive. However, we have observed a number of recent single-cell sequencing studies in HF, which reveal the change process of different cells during the pathological process through single-cell reconstruction^9^-^11^. In this study, we summarize those findings and detail the changes observed in immune cells, non-immune cells, and extracellular matrix components during HF development.

### 2.1 Immune cells

Immune cells, including monocytes/macrophages, neutrophils, T cells, B cells, mast cells, natural killer cells, and dendritic cells, are critical players in the inflammatory response during HF. Those cells exhibit dynamic changes in their populations, activation states, and interactions, which significantly influence cardiac function and remodeling.

#### 2.1.1 Monocytes and macrophage subtypes

**Figure 2:**
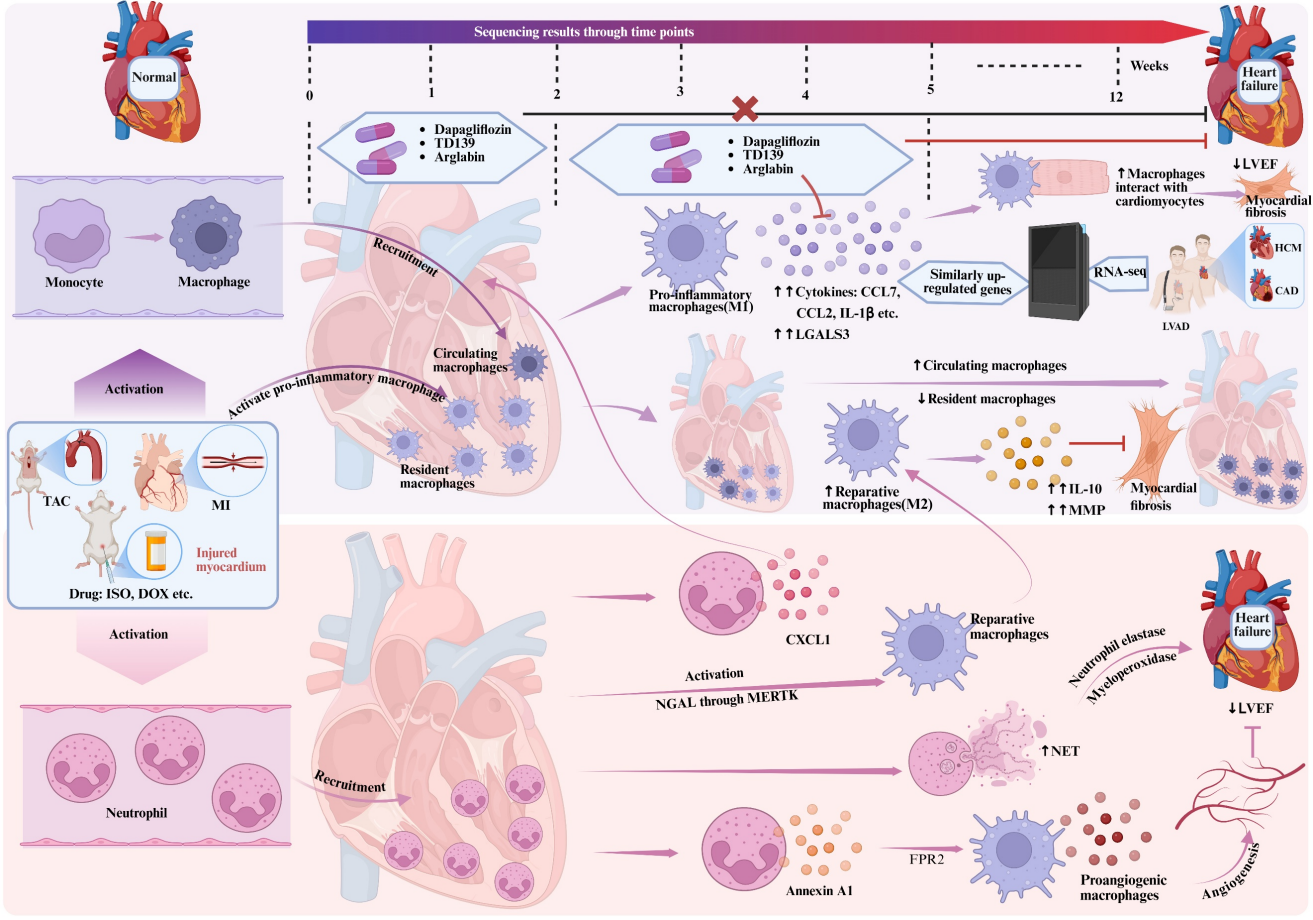
** Role of monocytes/macrophages and neutrophils in heart failure.** Figure 2 highlights the dynamic changes of monocytes/macrophages and neutrophils in the development of heart failure. As heart failure advances, circulating macrophages gradually replace resident cardiac macrophages. In addition, in the TAC model, treatments administered at 2-5 weeks, such as Dapagliflozin, TD139 and Arglabin, significantly preserved cardiac function and inhibited cardiac fibrosis at 5 and 8 weeks, whereas similar effects were observed at 0-2 weeks, indicating the effectiveness of specific targeting of macrophages in inhibiting pathological cardiac hypertrophy. Neutrophils, as key cells of the innate immune system, plays a significant role in the development of heart failure, being involved in the inflammatory response, cardiac remodeling, angiogenesis, and the repair process of cardiomyocytes, interacting with macrophages. CCL7, Chemokine (C-C motif) ligand 7; CCL2, Chemokine C-C motif ligand 2; IL-1β, Interleukin-1β; LGALS3, Galectin 3; LVEF, left ventricular ejection fraction; HCM, hypertrophic cardiomyopathy; CAD, coronary atherosclerotic heart disease; LVAD, left ventricular assist device; IL-10, Interleukin-10; MMP, matrix metalloproteinase; NET, neutrophil extracellular trap; FRP2, flavin reductase; NGAL, neutrophil gelatinase-associated lipocalin; CXCL1, C-X-C motif chemokine ligand 1; MERTK, mer tyrosine kinase.

In recent years, an increasing number of studies have highlighted the crucial role of macrophages in HF. Macrophages are not only involved in the innate immune response, but also related to cardiac contraction, action potential and angiogenesis^9^, ^10^, as shown in Figure 2. The dynamic changes of macrophages are particularly critical in the development of HF. Single-cell reconstitution of the adult heart during HF and recovery reveals the cellular landscape underlying cardiac function^9^. On the basis of gene ontology (GO) enrichment analysis, immune response-associated macrophages were found to be increased in patients with coronary heart disease compared to healthy individuals, while macrophages involved in electrical coupling were decreased. In addition, the number of macrophages did not change significantly even after left ventricular assist device (LVAD) treatment, regardless of whether the patients had partial recovery of cardiac function, indicating a persistent inflammatory state after LVAD treatment in end-stage HF, which may be related to the poor effect of LVAD treatment in some patients. In fact, inflammation can be both a cause and a consequence of HF, and it occupies a central position in the pathogenesis and progression of HF^4^.

In the progression trajectory of pathological cardiac hypertrophy, single-cell reconstruction reveals the cellular landscape of pathological cardiac hypertrophy^10^. It is found that after pressure overload-induced cardiac hypertrophy, cardiac function is progressively reduced in 2-5 weeks, while inflammation is increased, as manifested by significant enrichment of immune responses, cytokine and chemokine responses. At this stage, macrophages become the main altered cell type, with pro-inflammatory macrophages activated. The expression of galectin 3 (LGALS3) is significantly increased, and inflammatory factors such as chemokine (C-C motif) ligand 7 (CCL7), CCL2, and interleukin-1β (IL-1β) are also abundantly expressed. Compared to 2 weeks after transverse aortic constriction (TAC), the overall increase of cell-to-cell cross-talk was observed in 5 weeks after TAC, among which macrophage (MP0, MP4) -centered interactions, such as macrophage-cardiomyocyte interaction, change the most significantly^10^. With the development of inflammatory response and the depletion of macrophages, the percentage of resident macrophages gradually decreased in the 4th week until they were replaced by circulating derived macrophages in the 12th week^11^. RNA sequencing and gene set enrichment analysis using human heart samples showed that genes upregulated in macrophages in 5 weeks after TAC are significantly highly expressed in hypertrophic cardiomyopathy, confirming the similarity between mice and humans^10^. All in all, during the pathological myocardial hypertrophy, the differentiation of macrophages into pro-inflammatory morphology plays a key role in reducing cardiac function. Therefore, stage-specific targeting of macrophages may hold significant therapeutic potential in suppressing pathological cardiac hypertrophy. Experimental studies demonstrated that Dapagliflozin (sodium glucose transporters together 2 inhibitors), TD139 and Arglabin (two new anti-inflammatory agents) during the 2-5 weeks post-TAC intervention preserved cardiac function at both 5- and 8-week follow-ups, attenuated myocardial fibrosis, and significantly downregulated the expression of LGALS3, inflammatory cytokines (CCL6 and CCL24), and hypertrophic markers (NPPA and NPPB). However, no comparable effects were observed when administered during the first 2 weeks after TAC^10^. Recent studies have demonstrated that targeting the TLR4/MyD88 signaling pathway through specific inhibition reduces the polarization of pro-inflammatory macrophages and alleviates cardiac fibrosis^12^. Numerous preclinical studies have identified infiltrating pro-inflammatory macrophages and CCL2 receptor (CCR2) as promising therapeutic targets. Multiple strategies have been developed to inhibit the infiltration of pro-inflammatory macrophages into the heart, including CCR2 antagonists (e.g., BMS-741672, PF-04136309) and anti-CCR2 monoclonal antibodies (e.g., MLN1202, MLN1202)^6^, ^13^, ^14^. Those findings validate the efficacy of temporally precise macrophage modulation in mitigating pathological cardiac remodeling and underscore the critical importance of stage-specific therapeutic interventions in disease progression.

In conclusion, during HF, resident macrophages switch to an inflammatory state, interact with cardiomyocytes, and play a key role in reducing cardiac function. Subsequently, circulating derived macrophages are recruited to the heart to replace the exhausted resident macrophages and continue to participate in the immune response. Stage-specific administration of macrophage subtype switching has a certain effect on inhibiting pathological cardiac hypertrophy and preventing the decline of cardiac function, indicating the importance of precise intervention in the progression of the disease.

#### 2.1.2 Neutrophils

As a key cell of the innate immune system, neutrophils play an important role in the development of HF^15^, ^16^, as shown in Figure 2. They are not only involved in the inflammatory response, but also closely related to cardiac remodeling, angiogenesis and the repair process of cardiomyocytes^16^. In the early stage of HF, neutrophils, which is a vital part of the innate immune system, rapidly flood into the heart^17^. The main role of neutrophils is to eliminate dead cells and promote macrophages to polarize to a repair phenotype^18^. The rapid infiltration of neutrophils is closely related to the recruitment of circulation-derived monocytes/macrophages during cardiac remodeling^13^. Neutrophil-depleted mice develop impaired cardiac function, increased fibrosis, and progressive HF after myocardial infarction (MI), which is associated with altered macrophage polarization^18^. Oncostatin M, a cytokine produced by neutrophils and macrophages, induces dedifferentiated cardiomyocytes to secrete regenerating islet-derived protein 3β (REG3β), which fine-tunes cardiac healing by modulating macrophage infiltration dynamics in the injured myocardium^19^. Furthermore, neutrophils stimulate macrophage polarization toward a pro-reparative phenotype (e.g., enhancing phagocytosis of apoptotic cells) through MERTK-dependent signaling triggered by neutrophil-derived neutrophil gelatinase-associated lipocalin (NGAL)^18^. Those findings highlight the regulatory role of neutrophils in balancing inflammatory responses and orchestrating cardiac remodeling during heart failure progression.

However, the continuous increase of neutrophils may become an important pathogenic factor in chronic LV remodeling and ischemic cardiomyopathy^20^. Studies have shown that the persistence of neutrophils is directly related to left ventricular systolic dysfunction^21^. Induction of neutropenia alleviates cardiac hypertrophy and dysfunction, and reduces the influx of circulating monocytes/macrophages and activation of macrophages in the myocardium^17^. In HF, neutrophils produce a large amount of C-X-C motif chemokine ligand 1 (CXCL1) to recruit circulating monocytes/macrophages to the myocardium and participate in the process of cardiac hypertrophy and dysfunction^22^. Neutrophil NETosis (formation of neutrophil extracellular traps) is enhanced by neutrophils, as indicated by increased NET production by neutrophils and increased circulating NETs. NETs contain multiple toxic components, such as neutrophil elastase and myeloperoxidase (MPO), which may impair cardiomyocyte function, driving adverse LV remodeling and dysfunction^23^.

The activity of neutrophils extends beyond their role in inflammation and cardiac remodeling; they are also involved in angiogenesis. In a mouse model of MI, recruited neutrophils to the heart produce annexin A1, which promotes macrophage differentiation to a proangiogenic phenotype through FPR2 ligation, leading to vascular endothelial growth factor (VEGF)-A secretion, which facilitates angiogenesis in the ischemic heart^24^.

In conclusion, neutrophils play a multifaceted role in the dynamic changes of HF. They are not only involved in inflammatory responses and cardiac remodeling, but also affect cardiac healing and function through their interaction with macrophages. The activation and depletion of neutrophils have a direct impact on the progression and prognosis of HF, which provides an innovative perspective on HF management and uncovers a promising target for therapeutic intervention.

#### 2.1.3 T cells

T cells are a class of lymphocytes characterized by the expression of CD3. They can be divided into CD8^+^T cells and CD4^+^T cells according to the different surface molecules. While CD8^+^T cells primarily function as cytotoxic T cells that recognize and attack antigens binding to MHC class I molecules, CD4^+^T cells further differentiate into a variety of subtypes, including Th cells, which regulate the activity of B cells and other T cells, and Treg cells, which suppress immune responses^25^. In the context of chronic HF, T cell populations expand and become activated in the heart, and patients with HF have increased numbers of CD3^+^T cells in the myocardium, increased expression of CD3^+^T cells surface activation markers in blood, and enhanced adhesion to activated endothelial cells compared with healthy individuals^26^. In a mouse model of ischemic HF, CD4^+^ and CD8^+^T cells numbers are systematically increased, along with Th1, Th2, Th17, and Treg cell subsets in the heart, circulation, spleen, and mediastinal lymph nodes. Additionally, antigen-presenting dendritic cells are elevated in the heart and spleen^27^. Those changes suggest that T cells are activated in response to autoantigens and play a role in the progression of HF^28^. Of particular interest is the role of CD4^+^T cells in HF. In a mouse model of chronic HF, ablation of CD4^+^T cells alleviated pathological left ventricular remodeling, demonstrating the indispensable role of CD4^+^T cells in the progression of HF. Furthermore, transfer of splenic CD4^+^T cells or cardiac CD3^+^T cells from HF donor mice induced cardiac injury and left ventricular remodeling in recipient mice, further confirming that immune memory transfer of activated cardiac and splenic T cells is sufficient to cause cardiac injury^28^.

In HF, the role of Treg cells cannot be ignored. Compared with the control mice, HF Treg cells in mouse models substantially increase. However, despite this increase, the regulatory balance of Treg cells fails to provide sufficient anti-inflammatory effects to counteract tissue damage, Instead, those cells exhibit elevated levels of Th1 cytokines and TNF receptor 1 (such as interferons gamma), with the increasing number of anti-angiogenesis and promote fibrosis characteristics, while also resulting in a loss of immunoregulatory capacity^27^. Selective ablation of Treg cells can reverse left ventricular remodeling and dysfunction, reduce hypertrophy and fibrosis, and increase tissue neovascularization, suggesting that restoring normal Treg cell function may be a promising approach for therapeutic immunomodulation in HF^27^. T cells also play an important pathophysiological role in non-ischemic, pressure overload-induced HF. In a mouse model after TAC, T cells are recruited to the heart, and the numbers of CD4^+^ and CD8^+^T cells are increased^29^. Genetic models of T-cell deficiency and studies of antibody-mediated T-cell depletion have shown a central role for activated CD4^+^T cells in the progression of HF and the development of pathological hypertrophy, interstitial fibrosis, and left ventricular dysfunction^26^. Furthermore, the activation of T cells relies on specific antigen presentation, and studies suggest that blocking this signaling axis can slow the progression of heart failure, potentially representing a viable therapeutic approach to reduce the recruitment of cardiac Th1 cells in non-ischemic HF^29^.

**Figure 3:**
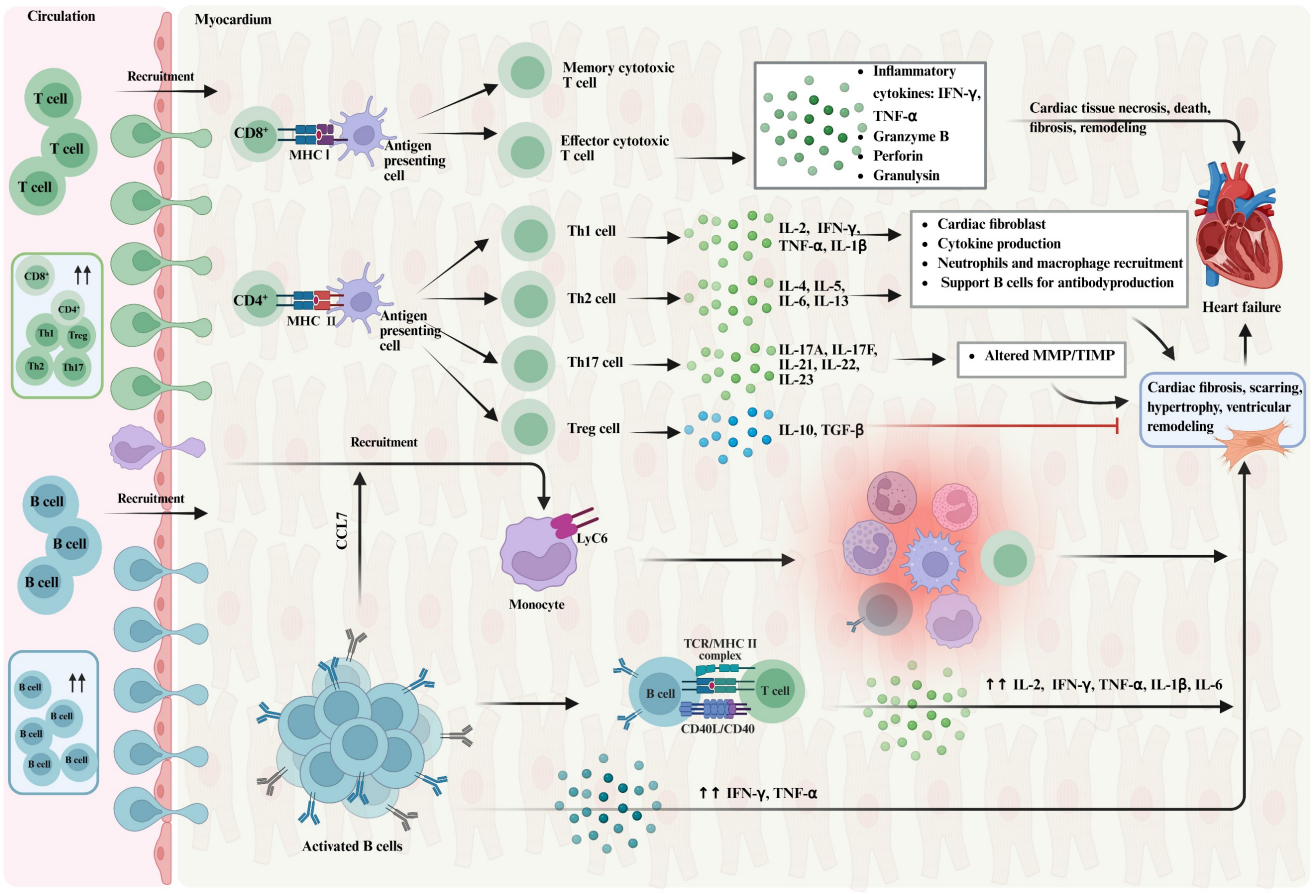
** Role of T cells and B cells in heart failure.** Figure 3 depicts the role of T cells and B cells in heart failure. T cells, characterized by the expression of CD3, are divided into CD8^+^T cells and CD4^+^T cells. CD4^+^T cells further differentiate into various subtypes, including TH cells and Treg cells, which regulate the activity of B cells and other T cells, and suppress immune responses. In chronic heart failure, T cell populations expand and become activated in the heart. B cells play a role in myocarditis, heart transplant rejection, and chronic myocardial inflammation and heart failure. The figure shows the changes in B cells and T cells, their interactions, and the impact on cardiac remodeling and function. IFN-γ, immune interferon γ; TNF-α, tumor necrosis factor-α; IL-2, Interleukin-2; IL-1β, Interleukin-1β;IL-4, Interleukin-4; IL-5, Interleukin-5; IL-6, Interleukin-6; IL-13, Interleukin-13; IL-17A, Interleukin-17A; Il-17F, Interleukin-17F; IL-21, Interleukin-21; IL-22, Interleukin-22; IL-23, Interleukin-23; IL-10, Interleukin-10; TGF-β, transforming growth factor-β; MMP, matrix metalloproteinase; TIMP, tissue inhibitors of metalloproteinase; CCL7, Chemokine (C-C motif) ligand 7;TCR, T cell receptor; MHC I, the major histocompatibility complex I; MHC II, the major histocompatibility complex II.

In conclusion, the role of T cells in HF is multifaceted, including promoting inflammation, regulating immune responses, and influencing cardiac remodeling and function, as shown in Figure 3. Those results offer fresh insights and potential targets for immunotherapeutic strategies in HF management.

#### 2.1.4 B cells

The role of B cells in the pathogenesis of HF has been gradually revealed. As a key component of the immune system, B cells are implicated in myocarditis and heart transplant rejection, while playing a pivotal role in chronic myocardial inflammation and HF. In the early stages of HF, the number and activity of B cells begin to change and assume a significant role in myocardial injury adaptation. Studies have shown that the number of B cells in the heart increases significantly after MI, potentially as a result of the recruitment of B cells from the bone marrow and spleen to the damaged heart area^30^. Natural IgM antibody secreted by B cells plays an important role in myocardial injury and selectively produces the chemokine CCL7, which induces the mobilization and recruitment of Ly6Chi monocytes to the heart, leading to tissue damage and deterioration of myocardial function in a mouse model of ischemia-reperfusion injury^31^. In addition, the activation of B cells plays an important role in chronic HF, and patients with dilated cardiomyopathy produce autoantibodies against a variety of cardiac proteins, which may lead to cardiomyocyte apoptosis and trigger progressive left ventricular dilatation and dysfunction^32^. In the progression of HF, the interaction between B cells and T cells, which can stimulate the production of pro-inflammatory cytokines, directly participate in cardiac remodeling by upregulating the cytokines transforming growth factor-β (TGF-β) and interleukin-6 (IL-6), and maintain a detrimental inflammatory environment by producing tumor necrosis factor-α (TNF-α), IL-1β, and IL-6^33^, cannot be ignored.

B cell depletion therapy has shown potential in the treatment of HF. B cell depletion can reduce left ventricular remodeling in a mouse model of myocardial ischemia-reperfusion injury, reduce myocardial hypertrophy and collagen deposition in AngⅡ-induced HF mice, and preserve left ventricular systolic function^34^. B cell deficient mice are protected from adverse LV remodeling after acute myocardial injury, which is mainly secondary to the reduced B cell mediated recruitment of Ly6C monocytes to the injured myocardium^35^. In clinical studies, B cell depletion therapy with rituximab improves cardiac function in patients with inflammatory dilated cardiomyopathy^36^. In a phase I/II trial involving patients with acute ST-segment elevation myocardial infarction, a single injection of rituximab (anti-CD20 monoclonal antibody) is being evaluated for B cell depletion^37^. This suggests that by regulating the number and activity of B cells, it may provide a new strategy for the treatment of HF.

Taken together, the dynamic changes of B cells in HF involve their recruitment, activation and interaction with other immune cells in the heart, as shown in Figure 3. Those changes affect the inflammatory response of the heart, and directly participate in cardiac remodeling and dysfunction. Therefore, a deep understanding of the mechanism of B cells in HF is vital for the advancement of innovative therapeutic strategies.

#### 2.1.5 Mastocytes

Mastocytes originate from the bone marrow, settle in the tissues after maturation, and participate in the storage and release of a variety of bioactive mediators, such as histamine, pro-inflammatory and anti-inflammatory cytokines, through their expression of KIT receptors^38^. In the heart, the number of mastocytes is increased in a variety of pathological conditions, including cardiac volume overload, chronic myocardial inflammation, and MI^39^. They are involved in regulating physiological processes such as vasodilatation, immune response and angiogenesis, while playing a driving role in the development of HF^40^.

Animal model studies have shown that mast cell activation and degranulation may promote adverse left ventricular remodeling in HF. For example, the study by Brower and Janicki found that pharmacological inhibition of mast cell degranulation attenuated left ventricular remodeling and improved survival in rats with chronic HF^41^. Furthermore, studies of the role of mastocytes in the progression of HF in KIT-deficient mice have shown that left ventricular remodeling and dysfunction are significantly reduced in KIT-deficient mice compared to wild-type mice^42^. This suggests that mastocytes may be involved in the pathological process of HF by regulating cardiac fibroblast activation and myocardial fibrosis.

In the heart of patients with HF, mastocytes are increased in density and are predominantly located in areas of interstitial fibrosis, expressing fibroblast growth factor 2 (FGF2), which may regulate fibroblast function in failing human hearts^43^. In addition, chymotrypsin secreted by mastocytes can convert angiotensin I to angiotensin II in the heart, a process that may be directly related to the development of angiotensin II-mediated fibrosis in HF^44^.

In summary, the role of mastocytes in HF is multifaceted, including promoting cardiac remodeling, regulating myocardial fibrosis, and affecting the angiotensin system. Those findings provide new potential targets in heart failure therapy, aiming to regulate mast cell activities and thereby enhance patient prognosis.

#### 2.1.6 Natural killer cells

As the major cellular effectors of the innate immune system, natural killer (NK) cells utilize germa-line encoded receptors to detect damaged or infected cells and decide whether to kill cells that come into contact based on the balance of signals from activated and inhibited receptors. However, to date, little is known about the role of NK cells in HF. In the context of HF, the number and activity of NK cells may change. It has been shown that circulating levels of CD16^+^CD56^+^ NK cells are significantly reduced in patients with HF with reduced ejection fraction (HFrEF), which is a significant difference compared to healthy individuals^45^. This reduced NK cell level was not associated with the etiology or severity of HF, suggesting that NK cell depletion may be a general immunomodulatory phenomenon. NK cells from HFrEF patients show diminished cytolytic function, which may affect their ability to clear damaged cells and regulate inflammatory responses^45^. This may be due to the effect of IL-6 on the responsiveness of NK cells to other cytokines through regulated signal transduction pathways, leading to the impairment of NK cell lytic function^45^. In addition, circulating levels of CD16^+^CD56^+^ NK cells were reduced in patients with PPCM during the early postpartum period, but NK cell levels gradually normalized in those patients over time^46^. This suggests that the dynamic changes in NK cells may be related to the specific stage of HF and the physiological state of the patient.

NK cells also interact with dendritic cells, macrophages, and T cells, stimulating the release of cytokines and chemokines from those cells, thereby mediating paracrine cytoprotection. This interaction may have important effects on cardiomyocyte apoptosis and cardiac remodeling^47^.

In summary, available evidence suggests that NK cells exhibit reduced number, impaired function, and interaction with other immune cells in HF. Those changes may have important effects on cardiomyocyte apoptosis, cardiac remodeling, and the progression of HF.

#### 2.1.7 Dendritic cells

The dendritic cells (DCs) not only bind and activate T cells through antigen presentation, but also regulate immune responses, inflammatory processes, and cardiac remodeling by secreting cytokines and growth factors^48^. In dilated cardiomyopathy (DCM), exogenously activated self-targeting DCs can trigger myocardial inflammation and DCM. In this process, DCs present the α-myosin peptide MYHCA-α, which activates CD4^+^ and CD8^+^ T cells, leading to T cell infiltration in myocardial tissue and impaired cardiac function^49^.

In animal models of acute myocardial injury, changes in DCs are closely related to the drive of T-cell-mediated inflammation and pump dysfunction after injury^50^. After coronary artery ligation, the density and activity of DCs were significantly increased, and the expression of costimulatory molecule CD86 was increased, initiating the activation of autoreactive IL-17^+^/IFN-γ^+^ T lymphocytes^51^. DCs alter the healing process after MI by activating T lymphocytes, producing inflammatory cytokines and directly activating fibroblasts^52^. They may also influence the development of inflammation by producing microRNA-rich exosomes^53^. However, the results of studies on DC depletion have been inconsistent, suggesting that the function of DCs may depend on the specific activation state of their subsets and the type of effector cells recruited. In some cases, depletion of conventional DCs has been associated with macrophage depletion after MI, followed by a reduction in infarct size, prevention of ventricular remodeling, and improved cardiac function^52^. However, in other studies, depletion of DCs has also been associated with a decrease in anti-inflammatory T-regulatory lymphocytes and impaired myocardial healing. Ideally, the activation of conventional DCs should be accompanied by an increase in the activity of tolerogenic DCs, with timely resolution of inflammation^52^. The mixture of cytokines IL-37 and troponin I leads to the generation of tolerogenic DCs, which has a beneficial effect on the process of remodeling after MI^54^. Local injection of tolerized DCs activates M2 macrophages via T regulatory lymphocytes and is beneficial for resolution of inflammation, wound healing, and ventricular systolic function^55^.

In the context of hypertension and diastolic HF (HFpEF), increased reactive oxygen species (ROS) production, isoprostane deposition, vascular collagen deposition, vascular inflammation, and renal dysfunction are associated with DCs^56^. DCs and isoprostanes are critical for the development of the hypertensive phenotype, and the accumulation of isoprostanes in DCs correlates with increased DC activity, as reflected by CD86 surface expression^56^. In addition, DCs act as drivers of T cell activation within the context of the hypertensive phenotype and possess the ability to stimulate T cell proliferation through their initiation^57^.

Therefore, the role of DCs in HF is multifaceted; they can either promote inflammation and cardiac injury, or protect the heart by modulating immune responses and promoting healing. Therefore, the activation state and function of DCs contribute significantly to the development and treatment of HF, offering innovative therapeutic targets and strategies in HF management.

In summary, the dynamic changes in immune cells during HF progression highlight their multifaceted roles in promoting inflammation, cardiac remodeling, and dysfunction. The population and interaction of specific immune cell offers some promising strategies to mitigate HF progression. Future research should focus on elucidating the precise mechanisms underlying those immune cell dynamics and developing targeted interventions to modulate their activity.

### 2.2 Non-immune cells

Non-immune cells, such as cardiomyocytes, endothelial cells, and fibroblasts, are also significantly impacted by inflammation and contribute to cardiac remodeling and dysfunction in HF.

#### 2.2.1 Cardiomyocytes

Cardiomyocytes (CMs) play a central role in the pathogenesis of HF, and their functional and structural changes directly affect the overall function of the heart. Single-cell sequencing has revealed the molecular heterogeneity of atrial and ventricular cardiomyocytes in heart tissue of HF, forming distinct subpopulations of uniquely characterized genes that reflect the specific functional requirements of different parts of the heart^9^. Atrial CM subsets are related to a variety of biological functions, while ventricular CM subsets are mainly involved in muscle development. Left ventricular CMs were enriched in oxidative phosphorylation and circadian rhythm related functions of myocardial contraction, while right ventricular CMs were enriched in protein processing signaling pathways in the endoplasmic reticulum. Such molecular differences may underlie the different functional requirements of the atrium and ventricle, and also reveal the complex changes of CMs in heart tissue in HF.

In different etiologies of HF, cardiomyocytes exhibit specific differentially expressed genes^9^, ^10^. S100A6 and PDK4 are up-regulated in coronary heart disease HF cardiomyocytes, while NPPA, NPPB and DKK3 are up-regulated in dilated cardiomyopathy HF cardiomyocytes. Changes in the expression of those genes may reflect individual mechanisms of disease progression. CHD-induced HF shows enrichment in processes related to protein targeting and energy metabolism, whereas dilated cardiomyopathy HF shows altered muscular system processes. In addition, TCF7L2 and CEBPD regulators showed reduced activities in both types of HF, while other regulators such as NR1H2, KLF4, XBP1, CREB5, EGR1 and JUN showed different regulatory features^9^.

Single-cell sequencing revealed that most cardiac cell subtypes changed dynamically over time during pathological cardiac hypertrophy. Overall, the expression of NPPA and NPPB was generally increased, while changes in structural and calcium handling genes were consistent with the stabilization and subsequent decline in cardiac function[10]The concomitant decreased expression of metabolic genes and induced expression of glycolysis-related genes suggest that cardiomyocytes may meet the enhanced energy demand through glycolysis during the early stage of cardiac hypertrophy^10^, ^58^. In addition, cardiomyocytes not only exhibit typical myocardial properties, but also highly express endothelial or fibroblast markers, such as Cdh5, Vwf or Vim, Dcn, involved in processes such as angiogenesis, antigen processing and presentation, or extracellular matrix (ECM) organization. Those functional changes and the dynamic changes of cardiac cell subtypes together affect cardiac remodeling and function.

#### 2.2.2 Endothelial cells

The role of endothelial cells (ECs) in HF is multifaceted, and they play an important role in the regulation of myocardial fibrosis, myocardial hypertrophy, cardiomyocyte contraction and angiogenesis. In HF, the endothelial cell regulates myocardial fibrosis through a variety of mechanisms^9^. When ECs are stimulated by physiological or pathological factors such as shear stress, pro-inflammatory factors, hypoxia, and so on, the related signaling pathways including transforming growth factor-β (TGF-β)/Smad, Notch, Wnt/β-catenin, and Ras are activated, thereby promoting the occurrence and development of EndoMT^59^. In addition, TGF-β1 secreted by ECs can promote the proliferation and migration of fibroblasts, activate the transformation of fibroblasts into myofibroblasts and secrete more extracellular matrix, thereby promoting myocardial fibrosis^59^.

ECs play a crucial role in regulating cardiac hypertrophy. Nitric oxide (NO), endothelin-1 (ET-1), neuregulin-1 (NRG-1), and other substances secreted by ECs act on cardiomyocytes, thereby influencing the process of cardiomyocyte hypertrophy^60^. NO affects cardiomyocyte hypertrophy by regulating the NO/sGC/cGMP signaling pathway. In HF with preserved ejection fraction (HFpEF), endothelial dysfunction can reduce the bioavailability of NO, leading to the blockage of the NO/sGC/cGMP pathway, and then cause cardiomyocyte hypertrophy and increased myocardial stiffness^61^. Endothelial cells also regulate cardiomyocyte contraction. NO secreted by endothelial cells can activate the NO/sGC/cGMP signaling pathway in cardiomyocytes, increase the activity of intracellular PKG, thereby reducing the sensitivity of myofilaments to calcium ions and inhibiting the contraction of cardiomyocytes^62^.

In the aspect of angiogenesis, endothelial cells are stimulated by related pro-angiogenic factors such as vascular endothelial growth factor, and the angiogenic signaling pathway is activated to promote angiogenesis^9^, ^58^. The mouse model of MI showed that the injection of atypical chemokine receptor 1^+^ (ACKR1^+^) ECs significantly reduced cardiac fibrosis and increased vascular density in the infarct and marginal zone^9^. The changes of specific EC subsets in the heart tissues of patients with ischemic cardiomyopathy are closely related to the pathological process of the heart^10^, ^58^. The expression of insulin receptor (INSR) is increased in EC-arterial cells of ischemic cardiomyopathy (ICM) patients, which may affect the signal transduction of angiogenesis. The expression of apoptosis regulatory gene XIAP-associated factor 1 (XAF1) is increased, and the expression of gelsolin (GSN) is decreased, which affects the balance of the survival and death of the cells. The expression of ​small mother against decapentaplegic protein (Smad6) is decreased, which may activate inflammation and fibrosis. In EC-angiogenic cells, INSR expression is increased and phosphatidylinositol-4,5-bisphosphate 3-kinase catalytic subunit type 2 alpha (PIK3C2A) expression is decreased, which affects the response and signaling of growth factors. Actin filament-associated protein 1-like 1 (AFAP1L1) expression is increased in EC-angiogenic cells and EC-general capillary cells, which may be related to the formation of actin stress fibers. The expression of multimerin 1 (MMRN1) is decreased in EC-lymphatic cells, which affects the function of lymphatic vessels.

Overall communication involving endothelial cells is increased in ischemic cardiomyopathy, particularly in endocardial and angiogenic endothelial cells. Cadherin-5 (CDH5), junctional adhesion molecule (JAM), laminin, and ​collagen signaling was significantly increased, which may be related to the extensive communication of endothelial cells with other cell types^58^, ^63^. Increases in laminin subunit alpha 4 (LAMA4), laminin subunit beta 1 (LAMB1), and ​laminin subunit gamma 1 (LAMC1) may have facilitated the attachment of endothelial cells to the basement membrane and affected the function of cardiomyocytes and other cardiac cell types^58^.

#### 2.2.3 Fibroblasts

In the early stages of HF, fibroblasts (FBs) have a decrease in the FB2 subset and an increase in the FB1, FB5, and FB4 subsets. Among them, the FB1 subset is associated with the extracellular matrix and shows functional overlap with ECs^9^. With the progression of HF, the proportion of FB subtypes with high expression of extracellular matrix proteins increases, and FB subtypes are largely involved in extracellular matrix and muscle cell development^10^. Differentially expressed genes (DEGs) showed increased cardiac fibrosis and chemokine gene expression in FBs during the middle and late stages of HF, indicating that changes in FBs are highly correlated with cardiomyocyte pathology^11^, ^58^. Gene set enrichment analysis on animals showed that genes up-regulated by FBs in the 11th week after TAC were the same genes that are highly expressed in HF patients, suggesting a potentially conserved cellular and molecular basis between mouse and human cardiac hypertrophy^10^.

At the end-stage of HF, cardiac fibroblast subpopulations marked by elevated expression of fibrosis-associated markers—specifically FB-thrombospondin 4, FB-fibronectin 1, and FB-periostin—demonstrate a progressive expansion correlating with disease severity^11^. This pathological trend is conserved across species, as evidenced by congruent observations in murine models of heart failure, highlighting the translational relevance of fibroblast activation dynamics in cardiac remodeling. Different FB subtypes in HF showed significant changes in constituent ratio. The proportion of FBs subtypes with enhanced angiogenesis, extracellular matrix and immune response regulation ability related to fibrosis development increased significantly^11^, ^58^. In summary, the dynamic changes of FBs in HF involve multiple aspects, including gene expression changes related to extracellular matrix (ECM) remodeling, cardiomyocyte dedifferentiation, angiogenesis, and immune response. Those changes were not only observed in mouse models but also confirmed in human HF samples, indicating that the FBs play a crucial role in the development and progression of HF.

In summary, non-immune cells, including cardiomyocytes, endothelial cells, and fibroblasts, exhibit dynamic changes in response to inflammation, contributing significantly to cardiac remodeling and dysfunction in HF. Targeting those cells and their interactions could provide therapeutic benefits by reducing fibrosis, improving cardiac function, and promoting tissue repair. Future research could focus on elucidating the molecular mechanisms underlying those non-immune cell dynamics and developing targeted interventions to modulate their activity.

### 2.3 Extracellular matrix

ECM not only provides structural support, but also promotes force transmission and transmits key signals to cardiomyocytes, vascular cells and interstitial cells to regulate the function of cardiomyocytes, and its changes play a key role in the pathogenesis of HF^64^. In HF, changes in the ECM are associated with the expansion of the cardiac interstitium and significant changes in its structural and biochemical composition^65^. Expansion of the cardiac ECM may contribute to diastolic dysfunction by increasing myocardial stiffness^66^. ECM deposition may contribute to systolic dysfunction by interfering with the excitation-contraction coupling of cardiomyocytes^67^. In patients with HF, expansion of the cardiac ECM is strongly associated with poor prognosis^68^. Changes in the ECM may also be involved in an increase in arrhythmic events and conduction abnormalities. ECM deposition disrupts the conduction of cardiac excitation and may play an important role in determining the arrhythmic nature of the failing heart^69^.

The pathophysiology of HF is remarkably heterogeneous, so the pattern of ECM changes and the relative role of matrix macromolecules in the pathogenesis of HF depend on the type and duration of myocardial injury^64^. For example, pressure overload triggers activation of early matrix synthesis programs, leading to expansion and activation of cardiac fibroblasts and synthesis of structural and nonfibrous ECM proteins^70^. Ischemic injury, on the other hand, leads to dynamic changes in the cardiac ECM that help regulate inflammation and repair, but may mediate adverse cardiac remodeling^71^. Persistent myocardial inflammation leads to temporal changes in the balance between matrix metalloproteinase (MMP) and TIMP (a tissue inhibitor of MMP) activity. The ratio of MMP to TIMP increases during episodes of inflammation, favoring degradation of the extracellular matrix^72^. However, the decrease of MMP/TIMP ratio after persistent inflammation, accompanied by the increase of mast cell-mediated TGF-β signaling, is conducive to progressive myocardial fibrosis^39^. It has also been found that mice with heart-specific overexpression of TNF exhibit defects in cardiac energy metabolism and progressive LV dilatation^72^. And TNF promotes LV dilatation by inducing the activation of MMP, which degrades extracellular collagen matrix^73^. In conclusion, the changes of ECM in different types of HF are heterogeneous, and understanding the role of ECM in them may help to identify therapeutic targets to reduce geometric remodeling and alleviate cardiomyocyte dysfunction.

The ECM undergoes significant remodeling during HF progression, contributing to myocardial stiffness, impaired cardiac function, and adverse clinical outcomes. Targeting ECM remodeling pathways offers a promising therapeutic strategy to mitigate fibrosis and improve cardiac function in HF. Future research should focus on elucidating the molecular mechanisms driving ECM changes and developing targeted interventions to modulate ECM remodeling.

The interplay among immune cells, non-immune cells, and the ECM forms a complex network that drives cardiac remodeling and dysfunction in HF. Immune cells initiate and sustain inflammation, while non-immune cells respond to inflammatory signals, contributing to fibrosis and remodeling. The ECM, in turn, provides a structural scaffold that influences cell behavior and cardiac function. Understanding those interactions is crucial for developing comprehensive therapeutic strategies that target multiple aspects of HF pathogenesis. Future research should focus on elucidating the precise mechanisms underlying those interactions and developing targeted interventions to modulate inflammation, fibrosis, and remodeling, ultimately improving HF outcomes.

## 3. Signaling pathway in the inflammatory of heart failure

### 3.1 MAPK signaling pathway

Mitogen-activated protein kinases (MAPKs) are a class of serine/threonine protein kinases that are highly conserved in cells and can respond to a variety of physical and chemical extracellular stimuli, such as inflammation, cytokines, and microRNAs, and transmit signals through tertiary cascades^74^. So far, MAPKs have been identified to include four subfamilies, namely ​extracellular signal-regulated kinase (ERK) family, c-Jun N-terminal kinase (JNK), p38/MAPK and atypical MAPK^74^. Those kinases are activated in turn and work together to regulate many important physiological and pathological effects, such as the proliferation, growth, and differentiation of cardiac resident cells such as cardiomyocytes, fibroblasts, endothelial cells, and macrophages^75^.

In HF, some molecules, transcription factors, or non-coding RNAs exacerbate oxidative stress and promote cardiac hypertrophy, apoptosis, and cardiac remodeling by phosphorylating the MAPK family. For example, I-κB kinase-ε (IKKε) binds p38, and UDP-glucose ceramide glycosyltransferase (UGCG) activates beta-1,4-galactosyltransferase 5 (B4GalT5) and Cyclophilin A (CyPA) induce reactive oxygen species (ROS) generation, all of which induce adverse reactions by phosphorylating ERK^76^-^78^. cardiotrophin-1 (CT-1) induces inflammation and cardiac fibrosis by increasing the level of Gal-3 through activation of ERK signaling pathway^79^. Arginine vasopressin (AVP) also initiates IL-6-mediated inflammatory response by activating ERK signaling pathway^80^. MiR-21 enhances ERK activity and induces fibrosis and cardiac hypertrophy by inhibiting sprouty homologue 1 (Spry1)^81^. In addition, overexpression of mammalian sterile 20-like kinase-1 (MST1) leads to JNK pathway activation, which initiates caspase-9-mediated cardiomyocyte apoptosis^82^. However, activation of the MAPK signaling pathway is not necessarily negative. ELABELA (ELA), a newly identified peptide with 32 amino acids, interacts with APJ to alleviate oxidative stress-induced apoptosis by phosphorylating ERK^83^.

### 3.2 TLR4/MyD88/NF-κB signaling pathway

Toll-like receptors (TLRs) are key components of the innate immune defense system, which can recognize pathogen-associated molecular patterns (PAMPs) and damage-associated molecular patterns (DAMPs). TLRs promote the transcription of pro-inflammatory cytokines by activating NF-κB through myeloid differentiation primary response factor 88 (MyD88)^84^. In HF, the activation of TLRs is closely related to the cardiac inflammatory response, among which TLR2 and TLR4 are the most reported. TLR2 expression is upregulated in cardiac myocytes, vascular endothelial cells, and resident immune cells in the HF model, whereas progression of HF is significantly reduced in TLR2^-/-^ mice^85^. Inhibitory treatment with anti-TLR2 antibody was able to block angiotensin II-induced cardiac fibrosis by inhibiting macrophage recruitment and inflammatory responses in the heart^86^. TLR4 expression is also upregulated in patients with chronic HF and induces the production of a large number of pro-inflammatory cytokines that promote the progression of myocardial dysfunction and fibrosis^87^. Pharmacological blockade of TLR4 alleviates myocardial ischemia/reperfusion (I/R) injury and the development of cardiac hypertrophy^88^.

TLR4/MyD88/NF-κB signaling pathway mediates inflammatory response, pyroptosis, oxidative stress and ion channel activity in HF, leading to cardiac dysfunction. Some molecules, transcription factors or non-coding RNAs are involved in the regulation. Previous studies have shown that bromodomain-containing protein 4 (BRD4) and alpha-amino-3-hydroxy-5-methyl-4-isoxazolepropionic acid (AMPA) activate the TLR4 signaling pathway to promote inflammatory response and oxidative stress-induced cardiac hypertrophy^89^. TLR4 signaling promotes macrophage polarization, pyroptosis and oxidative stress through activation of NLRP3 inflammasome and ​JNK^90^. MiR-340-5p can block TLR4 signal transduction by targeting high mobility group box 1 (HMGB1), thereby reducing inflammatory response^91^. In addition, TLR2 activation by heat shock protein-70 has a direct effect on the contractile performance of cardiomyocytes, while LPS activation of cardiac TLR4 increases Ca2^+^ efflux through sodium-calcium exchanger and action potential duration, leading to arrhythmic events^92^. Inhibition of the over-activation of TLR4/MyD88/NF-κB signaling pathway can reduce the damage of cardiomyocytes and protect cardiac function^93^, ^94^.

### 3.3 NLRP3/ caspase-1 signaling pathway

The NLRP receptor has attracted much attention due to its association with the inflammasome, the macromolecular protein complexes that mediate the inflammatory response under DAMP and PAMP signal transduction, and act by processing and activating pro-caspase-1 to become caspase-1^95^. Caspase-1 further activates a series of pro-inflammatory cytokines, such as IL-1β, IL-18 and high mobility group box 1 protein, which trigger a strong inflammatory response and aggravate the occurrence and development of HF^96^. Studies have shown that NLRP3 inhibition or knockout can significantly reduce inflammatory response, fibrosis and cardiac dysfunction^97^, ^98^.

In HF, the NLRP3/ caspase-1 inflammasome pathway is related to inflammation, pyroptosis, cardiac hypertrophy and fibrosis, and some molecules, transcription factors or non-coding RNAs are involved in the regulation. For example, Cardiomyocyte calmodulin-dependent protein kinase IIδ (CaMKIIδ) activates the NLRP3/ caspase-1 inflammasome pathway, leading to macrophage aggregation, cardiac fibrosis, and myocardial dysfunction^99^. ROS also up-regulates gasdermin D (GSDMD) by activating the NLRP3/ caspase-1 inflammasome pathway, leading to cardiac pyroptosis and cardiac hypertrophy^100^. MiRNA-223-3p and selective NLRP3 inflammasome inhibitor MCC950 inhibited NLRP3 signaling pathway to reduce inflammation, pyroptosis, cardiomyocyte hypertrophy and cardiac fibrosis^101^, ^102^. Inhibiting the excessive activation of NLRP3/ caspase-1 inflammasome can reduce the inflammatory response and myocardial fibrosis in HF^103^, ^104^.

### 3.4 JAK/STAT signaling pathway

Janus kinase (JAK) proteins are cytoplasmic tyrosine kinases that associate with the intracellular domain of membrane-bound receptors^105^. Its function is to transpose signals from extracellular ligands, such as cytokines and growth factors, to the nucleus to coordinate cellular responses^106^. Studies on the role of JAK/ signal transducer and activator of transcription (STAT) signaling pathway in cardiac tissue have mainly focused on STAT1 and STAT3^105^. STAT3-deficient mice had larger myocardial infarct size after ischemic injury, and the number of apoptotic cells was increased in the myocardium of rats with significantly inhibited STAT3 phosphorylation^107^. STAT1 induces apoptosis of I/R cells by up-regulating caspase-1, and silencing STAT1 can play a protective role in the heart^108^.

The JAK/STAT signaling pathway is essential for HF, apoptosis, immune regulation, and left ventricular remodeling, and some molecules, transcription factors, or non-coding RNAs are involved for HF, apoptosis, immune regulation, and left ventricular remodeling, and some molecules, transcription factors, or non-coding RNAs are involved^109^. MiR-181a and miR-150 inhibit the activation of JAK/STAT signaling pathway, attenuate the inflammatory response of DC and cardiomyocyte apoptosis^108^. SOCS1 overexpression is associated with a pronounced HF phenotype, possibly by persistent inhibition of glycoprotein 130 (gp130) signaling, involving the JAK/STAT signaling pathway^110^. Heart-specific deletion of SOCS3 inhibits myocardial apoptosis and fibrosis and prevents left ventricular remodeling after AMI14, which is associated with increased STAT3 expression^111^. However, long-term activation of STAT3 can affect remodeling after MI leading to HF^112^.

### 3.5 PI3K/Akt signaling pathway

PI3K, or phosphatidylinositol-3-kinase, is able to be activated by a variety of cell surface receptors, such as growth factor receptors and G protein-coupled receptors^113^. When activated, PI3K is able to convert phosphatidylinositol-4, 5-diphosphate (PIP2) to phosphatidylinositol-3,4, 5-triphosphate (PIP3), which acts as an activator of Akt/PKB (protein kinase B)^114^. Activation of Akt has important effects on cell survival, proliferation, metabolism and inflammatory response^115^.

In HF, the PI3K/Akt signaling pathway is closely related to inflammatory response, cardiomyocyte apoptosis, fibrosis, and cardiac function^116^. Akt regulates the inflammatory response by phosphorylating a variety of downstream target proteins, such as mTOR, glycogen synthase kinase-3 (GSK-3), ​forkhead box O (FOXO) family transcription factors and NOS^117^. For example, Akt affects the NF-κB signaling pathway by inhibiting the activity of GSK-3, thereby regulating the expression of inflammatory factors^118^. In addition, Akt can also reduce the expression of anti-inflammatory factors by phosphorylating FOXO, thereby aggravating the inflammatory response^119^. Some non-coding RNAs regulate inflammatory responses by targeting PI3K/Akt. For example, miR-144-3p inhibits cardiomyocyte apoptosis and improves myocardial injury by inhibiting PI3K/Akt axis by down-regulating suppressors of cytokine signaling 2 (SOCS2)^120^. Inhibition of the over-activation of PI3K/Akt signaling pathway can reduce the inflammatory injury of cardiomyocytes and protect cardiac function^119^, ^121^. Recent multi-omics analysis of BIOSTAT-CHF study also showed that PI3K/Akt activation was closely related to higher mortality in HF^122^. Therefore, the intervention strategy targeting the PI3K/Akt signaling pathway represents a potential novel approach for the treatment of HF^123^.

### 3.6 Wnt/β-catenin signaling pathway

Wnt is a growth stimulator that leads to cell proliferation, and the Wnt signaling pathway is associated with developmental processes^105^. Wnt activation is associated with pathological stages following myocardial injury, including inflammation, angiogenesis, and fibrosis^124^. On the one hand, Wnt-5a promotes the release of IL-1, IL-6, and IL-8 from monocytes and promotes the inflammatory response^125^. On the other hand, loss of Wnt inhibitor 1 (WIF1) leads to an increase in inflammatory monocytes and severe adverse remodeling, while overexpression of WIF1 attenuates monocyte responses and improves cardiac function^126^. In addition, pro-inflammatory macrophages activate the β-catenin mediated signaling pathway and increase the expression of inflammatory cytokines^127^. Inhibition of Wnt/β-catenin signaling can prevent excessive inflammatory response and cardiac dysfunction after MI^128^. In addition to inflammation, Wnt/β-catenin signaling pathway also plays a major role in the regulation of cardiac fibrosis^129^. The Wnt signaling pathway also interacts with TGF-β signaling. Wnt-3a can up-regulate TGF-β signaling through canonical β-catenin dependent Wnt signaling of Smad2 to induce myofibroblast differentiation^130^. MicroRNAs are also involved in the regulation of this signaling pathway. MiR-145 reduced cardiac fibrosis by directly targeting SOX9 in fibroblasts and regulating Akt/GSK-3β/β-catenin signaling pathway changes^131^.

### 3.7 TGF-β/Smad signaling pathway

As a pleiotropic cytokine, TGF-β affects the function and phenotype of cardiomyocytes, immune cells and vascular cells by binding to its downstream effector Smads, and participates in the repair and remodeling process after heart injury^132^. In the early stages of HF, the activation of TGF-β/Smad3 signaling pathway participates in the anti-inflammatory switch of macrophages by mediating the up-regulation of PPAR-γ and PPAR-δ, while promoting the synthesis of angiogenic and fibrotic signals and protecting the heart from structural damage^133^. However, long-term activation of TGF-β/ Smad3 signaling may lead to increased cardiac fibrosis and ventricular dysfunction^134^. In models of MI and HF, TGF-β promotes fibrosis through a Smad dependent pathway, while inhibition of TGF-β signaling reduces fibrosis and improves cardiac function^135^. Several molecules, transcription factors or non-coding RNAs promote cardiac hypertrophy, inflammation, fibrosis and remodeling by activating the TGF-β/Smad signaling pathway. For example, homeobox A5 (Hoxa5) induces cardiac hypertrophy by activating the TGF-β/Smad signaling pathway, which is inhibited by histidine triad nucleotide-binding protein 1 (HINT1)^136^. Cardiac fibroblasts inhibit TGF-β/Smad3/IGFBP7 signaling pathway through Htra3 to reduce inflammation, fibrosis, and improve cardiac dysfunction^137^. Notably, in a recent study, single cell sequencing identified six TGF-β-related hub genes (TANC2, ADAMTS2, DYNLL1, MRC2, EGR1 and OTUD1), all of which showed abnormal trends in cardiac hypertrophy^138^. In summary, the mechanism of TGF-β/Smad signaling in HF involves multiple levels, including promoting fibrosis, regulating immune response and affecting vascular stability. Those effects may be either protective or lead to further deterioration of cardiac function. Therefore, therapeutic strategies targeting TGF-β/Smad signaling require precise regulation to ensure that adverse remodeling is inhibited without interfering with cardiac repair and protective mechanisms^139^.

### 3.8 RhoA/ROCK signaling pathway

RhoA is one of the most important members of the Rho family, whose main function is to regulate the actin cytoskeleton in eukaryotes. The spatiotemporal regulation of RhoA activation is responsible for cell morphology, adhesion, and cell motility^105^. ROCK is a key downstream target of RhoA. The RhoA/ROCK signaling pathway regulates cardiomyocyte survival, oxidative stress, cardiac hypertrophy and fibrosis^140^. Hif1α is a major regulator of hypoxia response and participates in myocardial fibrosis^141^. Studies have found that as an upstream pathway in multiple pathophysiological processes of Hif1α, RhoA/ROCK signaling pathway mediates Hif1α to promote myocardial fibrosis and apoptosis, and this process is negatively regulated by Notch3^142^. Recent studies have also shown that LIM domain kinase 1 (LIMK1), a serine/threonine kinase, which controls actin polymerization by phosphorylation and inactivation of cofilin, Activation of RhoA/ROCK signaling pathway induces inflammatory response, oxidative stress, apoptosis and cardiac remodeling in HF rats, and this process is negatively regulated by miR-93^143^.

### 3.9 Notch signaling pathway

Notch signaling is a key mechanism of intercellular communication in ischemic and fibrotic diseases, and plays a protective role in cardiac ischemia by reducing oxidative stress and myocardial injury induced by reperfusion^144^. In the context of cardiac pressure overload, lack of Jagged1 in cardiomyocytes leads to cardiac hypertrophy and fibrosis, while Notch activation inhibits myofibroblast proliferation^145^. Notch signaling plays a crucial role in maintaining the endothelial phenotype^146^. Inhibition of Notch signaling, particularly in the endothelium of adult mice, leads to cardiac hypertrophy and failure^147^. Notch inhibitors promote EndMT and cardiac fibrosis, and activation of Notch signaling protects the heart^148^. Recent studies have found that abnormal expression of aldolase A (ALDOA) activates the Notch1/Jagged1 signaling pathway by upregulating VEGF to alleviate oxidative stress injury in cardiomyocytes^149^. In addition, Notch1 can also inhibit the downstream apoptosis-related pathway by activating neuregulin 1 (Nrg1), and reduce the apoptosis induced by DCM^150^. In the in vitro experiment of hypoxic cardiomyocytes model, Notch1 also regulated cell apoptosis by down-regulating Bcl-2 and Bax and up-regulating caspase-9 and -3^151^.

### 3.10 cGAS/STING signaling pathway

Cyclic guanosine monophosphate-adenosine monophosphate synthase (cGAS) is a cytoplasmic DNA sensor that is activated upon binding to double-stranded DNA (dsDNA), which then activates stimulator of interferon genes (STING) signaling^152^. The activation of cGAS-STING signaling pathway is involved in a variety of cellular processes such as inflammation, autophagy, and apoptosis in HF^153^. In TAC-induced HF, the cGAS-STING signaling pathway is activated, and inhibition of this pathway can down-regulate the early inflammatory response of TAC, reduce myocardial hypertrophy, fibrosis and pyroptosis, and preserve myocardial contractility^154^. In addition, cGAS-and STING-deficient mice reduced myocardial hypertrophy, inhibited inflammatory cell infiltration and inflammatory response, reduced cardiomyocyte apoptosis, and improved survival^155^. STING inhibitors also produced similar effects^156^. Clinical studies have shown that the use of a left ventricular assist device (LVAD) decreases cGAS production in patients with ischemic cardiomyopathy^153^. However, activation of the cGAS-STING signaling pathway is not necessarily negative. Studies have found that STING overexpression can significantly reduce cardiac hypertrophy, fibrosis and inflammation, and improve cardiac function^157^. HF and cGAS-STING signaling pathway have attracted increasing attention. Targeting cGAS-STING signaling pathway may be beneficial to the treatment of HF, but further studies are needed to determine its exact relationship with disease progression.

### 3.11 NRF2 signaling pathway

NRF2 is a product of NFE2L2 gene, which is important for maintaining ROS homeostasis and participating in the regulation of antioxidant genes^158^. Heme oxygenase (HO) is a rate-limiting enzyme that catalyzes the production of Heme produces biliverdin Ixα, carbon monoxide (CO) and iron from heme. As a downstream target of NRF2, HO is involved in anti-oxidative stress and cell protection^105^. NRF2 knockout mice had left ventricular diastolic dysfunction, cardiac hypertrophy and down-regulation of Sarcoplasmic/endoplasmic reticulum Ca2^+^ ATPase 2a (SERCA2a), as well as increased β-MHC, ANF and BNP mRNA levels in myocardium^159^. Suggesting that NRF2 deficiency leads to rapid progression of HF^160^. NRF2 up-regulation reduced oxidative stress and fibrosis caused by RKIP^-/-^ mice^161^. In H9C2 cells, silencing BRD4 reduced inflammatory response, oxidative stress and cardiac hypertrophy by activating NRF2/HO-1^162^. NRF2/HO-1 also protects cardiomyocytes from oxidative stress by regulating ion channels^105^. However, a number of clinical and basic studies have found that NRF2 expression is up-regulated in HF, which may be related to the inhibition of NRF2 translation^163^. Studies have shown that the down-regulation of NRF2 protein expression reduces the antioxidant enzymes it targets and increases NRF2 transcription within 6 weeks after MI, suggesting that translational inhibition of NRF2 may contribute to the dysregulation of HF^164^. Further studies showed that the up-regulation of local miR-27a, miR-28-3p and miR-34a induced by MI may inhibit NRF2 translation and promote oxidative stress and cardiac remodeling. Therefore, upregulation of Nrf2/HO-1 axis has a cardioprotective effect^165^, ^166^.

### 3.12 Hippo signaling pathway

The Hippo pathway is an evolutionarily and functionally conserved signaling pathway that controls organ size by regulating cell proliferation, apoptosis, and differentiation. The major signaling output of the Hippo pathway is through the transcriptional co-regulator yes-associated protein (YAP), which coordinates with transcription factors such as TEA domain family member 1 (TEAD1) to regulate the expression of numerous target genes^167^. Abnormal Hippo pathway activity, such as elevated levels of phosphorylated YAP and LATS, has been detected in heart samples from ischemic or non-ischemic HF patients, indicating that Hippo signaling pathway plays a regulatory role in the pathological process of myocardial ischemia followed by HF^168^. Activation of Hippo signaling can promote the development of dilated cardiomyopathy (DCM) by inhibiting mitochondrial gene expression and mediating mitochondrial damage^169^. Studies have shown that inflammatory cells, cardiac fibroblasts and vascular endothelial cells also play a crucial role in regulating cardiac function through the Hippo/YAP pathway during the recovery from myocardial injury^170^. On the other hand, Hippo pathway kinase MST1 plays an important role in I/R and MI, and this role is independent of YAP/TAZ. Oxidative stress induced by I/R or MI can activate MST1 in mitochondria, thereby inhibiting cardiac autophagy and promoting cardiomyocyte apoptosis^171^. Cardiomyocyte-specific overexpression of MST1 can lead to dilated cardiomyopathy and increase cardiomyocyte apoptosis, while overexpression of dominant negative MST1 (which inhibits endogenous MST1) can significantly reduce I/ R-induced apoptosis^172^.

## 4. Clinical association between inflammation and heart failure

### 4.1 Relationship between inflammation and cardiac function in heart failure

**Figure 4:**
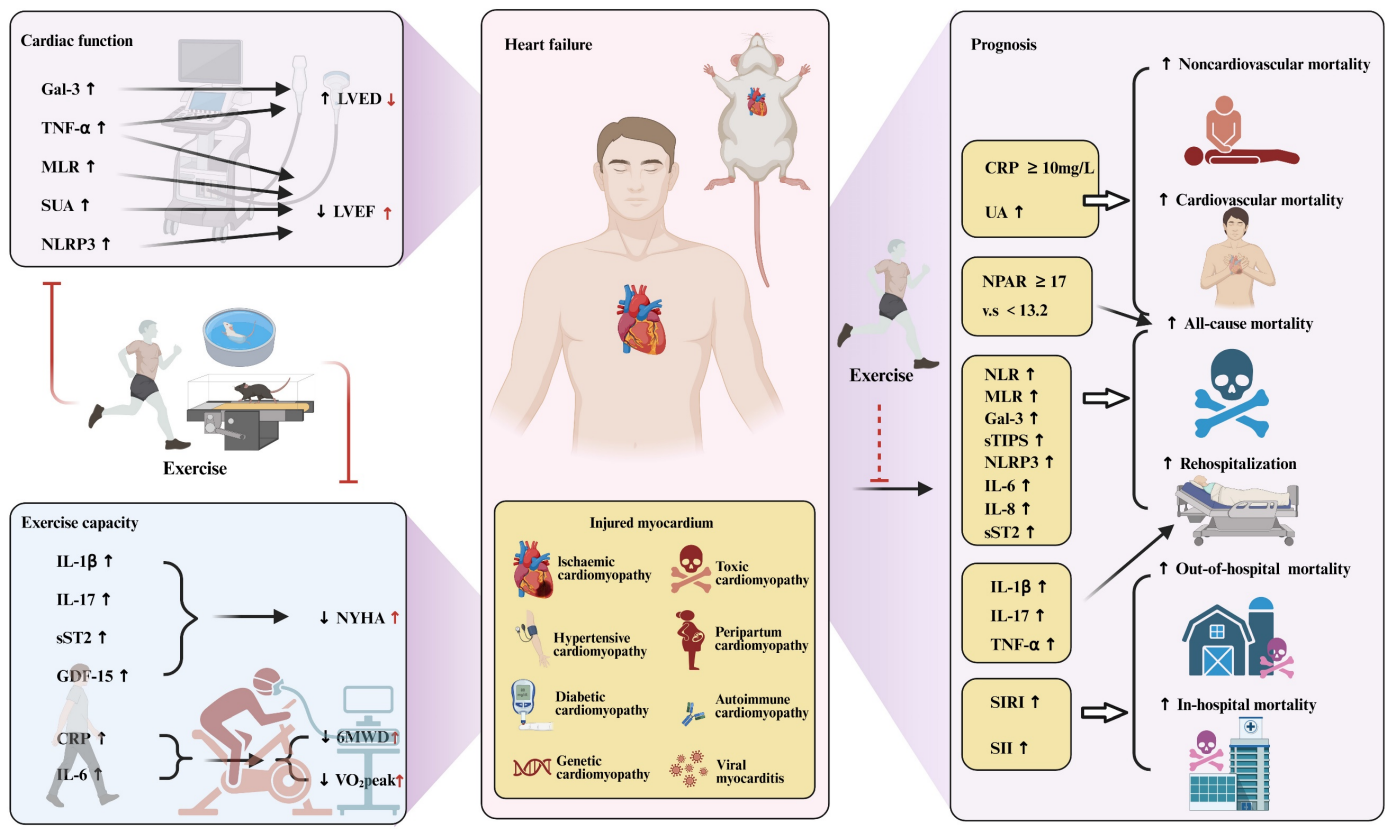
** Heart failure, inflammation and exercise.** Figure 4 explores the relationship between heart failure, inflammation, and exercise. It demonstrates how exercise intervention may improve cardiac function in heart failure by modulating the inflammatory response. Exercise intervention may alleviate the inflammatory response by reducing the activity of pro-inflammatory cytokines, and improve cardiac function by reducing myocardial fibrosis, oxidative stress, and apoptosis. Gal-3, Galectin 3; TNF-α, tumor necrosis factor-α; MLR, monocyte-to-lymphocyte ratio; SUA, serum uric acid; NLRP3, pyrin domain-containing protein 3; LVED, left ventricular end-diastolic diameter; LVEF, left ventricular ejection fraction; IL-1β, Interleukin-1β; IL-17, Interleukin-17; sST2, soluble suppression of tumorigenesis-2; GDF-15, growth differentiation factor 15; CRP, C-reactive protein; IL-6, Interleukin-6; NYHA, New York Heart Association; 6MWD, the 6-minute-walk distance; VO_2_peak, peak oxygen uptake; NPAR, neutrophil-to-albumin ratio; NLR, neutrophil-to-lymphocyte ratio; sTIPS, simplified thrombo-inflammatory score; IL-8, Interleukin-8; SIRI, systemic inflammation response index; SII, systemic immune inflammatory index.

The inflammatory response can be either a cause or a consequence of HF and plays a central role in the pathogenesis and progression of HF^4^. Clinical studies have shown that systemic inflammatory markers have a certain correlation with cardiac function in patients with HF, as shown in Figure 4. The first is cardiac structure and function. Galectin-3 (Gal-3) levels are positively correlated with an increase in left ventricular end-diastolic volume^173^. In addition, increased TNF-α levels were associated with left atrial dysfunction and left ventricular diastolic and systolic dysfunction^174^. The monocyte-to-lymphocyte ratio (MLR) was also closely related to LVEF^175^. However, soluble suppression of tumorigenesis (sST2) levels did not correlate with LV structure or LV systolic or diastolic function^176^. In addition, a strong inverse relationship was found between serum uric acid (SUA) and LVEF in HF patients, independent of renal function and diuretic use^177^.

This was followed by the New York Heart Association (NYHA) classification. The current study showed that the NYHA classification of HF increased with increasing levels of inflammatory factors such as IL-1β, IL-17, sST2, and GDF-15. In humans, IL-1β has been shown to be directly proportional to NYHA functional status, regardless of etiology^178^. Elevated IL-17 levels have been shown to correlate with the NYHA classification of HF, meaning that higher levels of IL-17 indicate more severe NYHA classification^179^. sST2 levels were significantly correlated with NYHA classification^180^. GDF-15 levels are significantly increased in patients with HF, and there is a clear relationship between GDF-15 levels and NYHA stage, that is, the higher the GDF-15 level, the more severe the NYHA grade^181^.

In addition, inflammation also has a certain impact on exercise capacity in patients with HF. The 6-minute walk test (6MWT) and cardiopulmonary exercise testing (CPET) are two important tests used to objectively assess exercise capacity and cardiopulmonary fitness in patients with HF. The main measurements are 6-minute walk distance (6MWD) and peak oxygen uptake (VO_2_peak)^182^, ^183^. Studies have found that changes in C-reactive protein (CRP) levels are associated with functional exercise capacity in patients with HF, and increased CRP levels are associated with decreased 6MWD and VO_2_peak^184^. Similarly, IL-6 levels were associated with motor function in HF patients, with HF patients with increased IL-6 levels showing lower peak VO_2_peak and 6MWD^185^. In conclusion, existing studies have shown that inflammation affects both dynamic and static cardiac function performance in HF.

### 4.2 Predictive role of inflammation in heart failure risk and prognosis

Current studies have shown that systemic inflammatory markers are associated with poor prognosis in patients with HF, as shown in Figure 4. A number of studies have shown that higher CRP levels are associated with poor prognosis in patients with HF^181^, ^186^. When the baseline CRP level was ≥10mg/L, the risk of all-cause death, cardiovascular death and non-cardiovascular death increased by 1.49 times, 1.26 times and 1.96 times, respectively^186^. Systemic inflammatory response index (SIRI) and systemic immune inflammation index (SII) are associated with the risks of in-hospital and long-term mortality in patients with HF, and the risk of all-cause death increases with the increase of SIRI and SII^187^-^189^. Neutrophil-to-lymphocyte ratio (NLR), MLR, and simplified thrombotic inflammation score (sTIPS) are associated with mortality and rehospitalization in HF patients^190^-^193^. In contrast, the prognostic role of PLR in HF outcomes is less clear, and higher PLR is associated with poor clinical outcomes in AHF patients but is not an independent predictor of long-term mortality in HF^192^, ^194^-^196^. Neutrophil percentage to albumin ratio (NPAR) is a relatively new marker that proved to be a potentially useful prognostic indicator of mortality in patients with HF in the intensive care unit and in the community^196^, ^197^. Individuals with elevated NPAR≥17 (vs.<13.2) had an 81% increased risk for all-cause mortality, and those with elevated NLR≥3.3 (vs.<1.7) had a 59% increased risk for mortality^196^.

In addition to systemic inflammatory markers, pro-inflammatory cytokines also play an important role in the prognosis evaluation of HF. IL-1β, IL-17 and TNF-α are independent predictors of HF readmission in HFpEF patients^198^. IL-6, IL-8, and sST2 were independently associated with increased risk of death or HF hospitalization^198^, ^199^. GDF-15 has also been extensively studied and identified as a prognostic factor for cardiovascular and all-cause mortality in HF^200^, ^201^. Circulating levels of NLRP3 inflammasome were associated with cumulative rehospitalization rates at 6 months^202^.

Gal-3 is also a predictor of death and rehospitalization in HF, but its prognostic ability in HF is less robust than that of NT-proBNP or sST2^203^, ^204^. The sub analysis of RELAX-AHF and PROTECT studies did not show the predictive value of Gal-3 for 6-month mortality, which may be due to the fact that the prognostic accuracy of Gal-3 in HF is affected by many factors^205^. In addition, in recent years, several studies have shown that elevated UA level is a predictor of poor prognosis in patients with HF, which is associated with an increased risk of all-cause death, cardiovascular death, and a composite endpoint of death or cardiac events^206^, ^207^. A meta-analysis of 18 studies with a sample size of more than 33,000 showed that UA was positively associated with the risk of all-cause death, cardiovascular death, and combined death or cardiac events in patients with HF^207^.

## 5. Exercise, heart failure and inflammation

### 5.1 Effect of exercise in heart failure

Exercise can improve exercise endurance and quality of life in patients with HF, as shown in Figure 4. It is a treatment strategy to reduce the number of repeated hospitalizations and is recommended by relevant guidelines^7^. Exercise improved cardiac function (e.g., LVEF) and motor function (e.g., VO_2_peak) in patients with HF, and there was no difference between groups in different degrees of HF^208^. In addition, the HF-ACTION study showed that exercise can improve patients' health status, and the beneficial effects of exercise rehabilitation are independent of the patients' pre-exercise endurance level. Even patients with HF who have a high exercise tolerance level still benefit from exercise rehabilitation^209^. A randomized clinical trial of 123 patients with HF completed over 10 years also showed that exercise rehabilitation could improve patients' exercise tolerance, ventilation efficiency and health status without increasing the incidence of major adverse events^210^. A study of 80 patients with HF who underwent aerobic exercise rehabilitation for 4 months and followed up for 1 year showed that there was a significant improvement in exercise function and quality of life after 4 months and 1 year of follow-up, indicating that aerobic exercise rehabilitation has short-term and long-term effects on improving exercise function and quality of life in patients with HF^211^. Multiple meta-analyses have shown that exercise rehabilitation can significantly improve quality of life and physical function, independent of study design, study quality, participant demographics, and disease severity^212^, ^213^. However, inflammation levels influence the effectiveness of exercise in HF patients^214^. Exercise significantly improved exercise capacity in patients with low inflammatory biomarkers, while improvement in VO_2_peak after exercise was slower in patients with high inflammatory biomarkers, indicating that inflammation affects motor function performance in HF patients.

In addition, studies have shown that exercise can moderately reduce the risk of clinical adverse events and reduce the risk of death and rehospitalization in HF patients^210^, ^215^. Multi-center clinical studies have shown that exercise can reduce the composite end point of cardiovascular death and hospitalization for HF, and the composite end point of all-cause death and hospitalization after adjusting for relevant factors^215^. Some scholars have also found that exercise can reduce the risk of HF readmission and cardiovascular death in patients with HF after 10 years of follow-up^210^. Meta-analysis shows that exercise rehabilitation can reduce the risk of all-cause and HF rehospitalization in the short term, and improve the risk of all-cause death in HF patients in the long term^213^. However, several recent meta-analyses, including the Meta-analysis of 25 randomized controlled trials involving 4481 patients with HFrEF and the meta-analysis of 18 clinical trials involving 3912 patients with HFrEF, have shown that exercise has no significant effect on the risk of death and rehospitalization in patients with HF^216^-^218^. In addition, the European Society of Cardiology guidelines also pointed out that there is a lack of strong evidence to prove that exercise rehabilitation is beneficial to the survival of patients with HF^219^. Therefore, the effect of exercise rehabilitation on the prognosis of patients with HF needs to be further studied.

### 5.2 Potential mechanisms of exercise in the intervention of inflammation in heart failure

**Figure 5:**
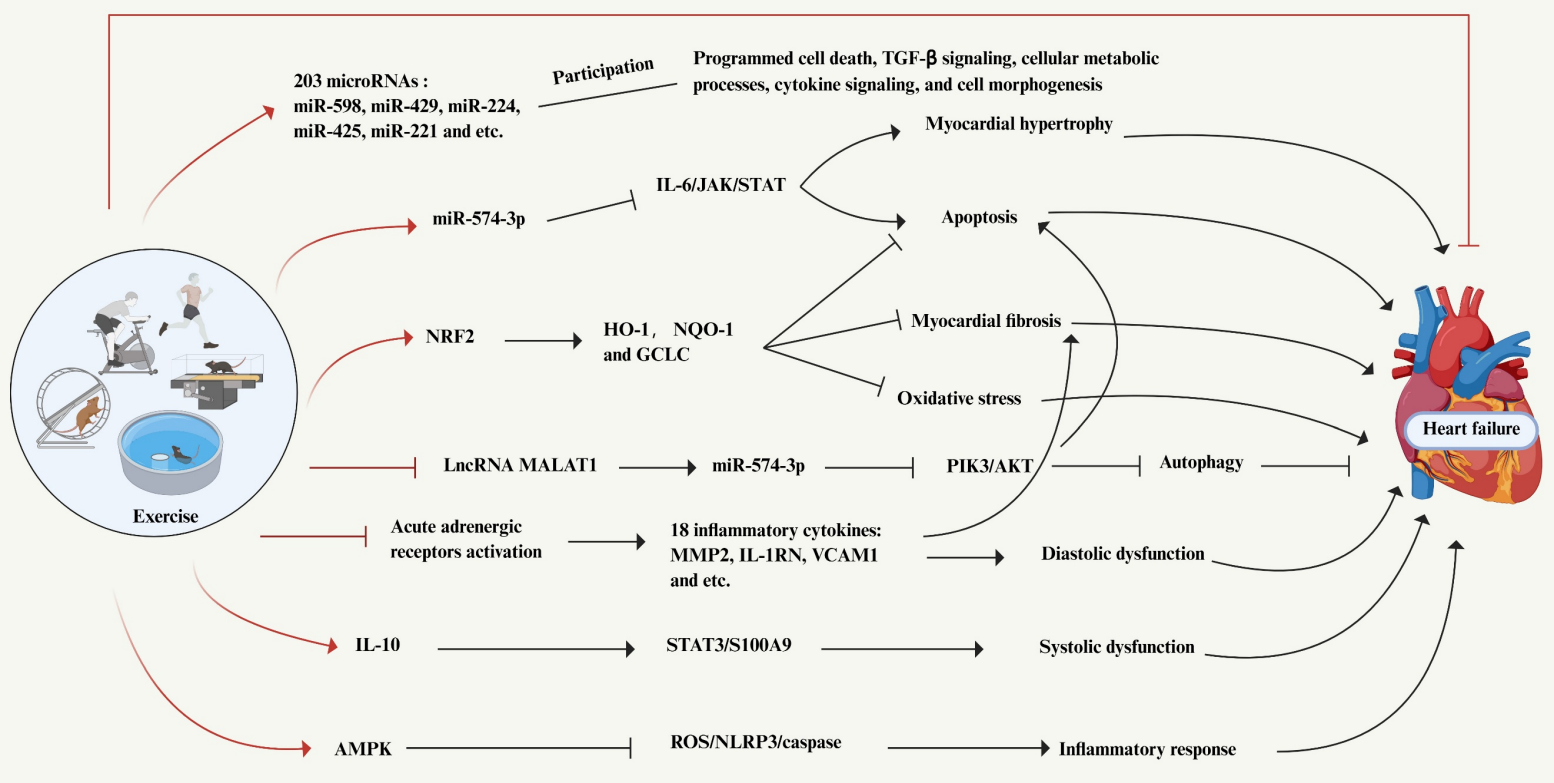
** Potential mechanisms of exercise in the intervention of inflammation in heart failure.** Figure 5 illustrates the potential mechanisms by which exercise intervention may regulate the inflammatory response in heart failure. Exercise may reduce the symptoms and progression of heart failure by modulating inflammatory mediators in the heart and circulation. The figure outlines the complex anti-inflammatory mechanisms of exercise, involving multiple molecules, transcription factors, and non-coding RNAs, and the regulation of signaling pathways such as JAK, NLRP3 and PI3K. TGF-β, transforming growth factor-β; IL-6, Interleukin-6; JAK, Janus kinase; STAT, signal transducer and activator of transcription; NRF2, NF-E2-related factor 2; HO-1, heme oxygenase-1; NQO-1, an quinone oxidoreductase 1; GCLC, glutamate-cysteine ligase catalytic subunit; MALAT1, metastasis-associated lung adenocarcinoma transcript-1; PIK3, phosphatidylinositol 3-kinase; AKT, threonine-serine protein kinase; MMP2, matrix metalloproteinase 2; IL-1RN, Interleukin-1RN; VCAM1, vascular cell adhesion molecule 1; S100A9, S100 Calcium Binding Protein A9; ROS, reactive oxygen species; NLRP3, pyrin domain-containing protein 3.

"Sterile" systemic chronic inflammation is involved in the development and progression of HF and is characterized by long-term (months to years) activation of immune components and excessive production of inflammatory cytokines^6^, ^220^. Recent research trends indicate that the anti-inflammatory effect of exercise as an intervention may play a role in delaying the progression of HF, which has gradually attracted attention, as shown in Figure 5. Despite this, our understanding of how exercise affects the inflammatory response in HF through anti-inflammatory mechanisms is still limited. An exercise induced proteomic profile change in advanced HF patients revealed that in CPET, 86 proteins in peripheral plasma of HF patients showed differences, which were mainly involved in immune response and inflammatory processes, coagulation, cell adhesion, regulation of cellular response to stimuli, and regulation of programmed cell death^221^. The results of changes in proteomic signatures reveal the complexity of exercise induction in HF patients, and also highlight the need for in-depth research on the specific biological pathways underlying those changes. Another report on the regulation of cardiac microRNAs induced by exercise training during HF showed that 17 microRNAs were particularly highly expressed in the exercise group compared with the sedentary group rats, including miR-598, miR-429, miR-224, miR-425 and miR-221, among others^222^. They also constructed a microRNA-mRNA regulatory network, which revealed a set of 203 microRNA target genes involved in programmed cell death, TGF-β signaling, cellular metabolic processes, cytokine signaling, and cell morphogenesis. Together, the results suggest that exercise alleviates cardiac abnormalities during HF by modulating cardiac microRNAs, which may play a potential role in cardioprotective mechanisms through multiple effects on gene expression.

Clinical studies have shown that on the basis of simvastatin treatment, exercise can reduce inflammation, reduce myocardial injury and adverse cardiac events, and improve cardiac function. The possible mechanisms are related to increasing MMP expression in peripheral lymphocytes, activating JAK/STAT3 signaling pathway, reducing mitochondrial damage, and inhibiting inflammatory response^223^. Basic studies have shown that exercise can promote the translocation of STAT3/S100 calcium-binding protein A9 (S100A9) to the nucleus by stimulating the secretion of interleukin-10 (IL-10) from macrophages. It can regulate the differentiation of Myeloid-derived suppressor cells (MDSC), increase the number of MDSCs, reverse isoproterenol (ISO) -induced cardiac dysfunction, and then prevent HF^224^. The MDSC is a kind of immunosuppressive cell, and its main role is to suppress immune response. A large amount of evidence has confirmed that MDSC has an inhibitory effect on T cells^225^, ^226^. Interestingly, this study found that exercise protected the heart by increasing MDSC numbers, but not by suppressing T cells. Exercise also attenuated ISO-induced macrophage infiltration and cardiac inflammation by inhibiting ROS/NLRP3/caspase-1 signaling pathway in an AMPK-dependent manner^227^. In addition, some studies have found that exercise prevents the development of HF by inhibiting the up-regulation of 18 inflammatory cytokines, such as MMP2, IL-1RN, and VCAM1, after acute adrenergic receptor overactivation, and reducing cardiac fibrosis and diastolic dysfunction^228^.

Exercise alleviates cardiac fibrosis and diastolic dysfunction by stimulating endothelial cells to secrete FSTL1 and activating TGFβ-Smad2/3 signaling pathway in MI rats^229^. Interestingly, Akt and ERK1/2 signaling pathways were also enhanced in the myocardium of rats after exogenous FSTL1 administration, indicating that multiple signaling mechanisms may be involved in FSTL1-mediated cardioprotection in MI rats, which deserves further elucidation. In contrast, exercise has been found to increase Fibroblast growth factor 21 (FGF21) protein expression, inactivate the TGF-β1-Smad2/3-MMP2/9 signaling pathway, reduce myocardial fibrosis, oxidative stress and apoptosis, and finally improve cardiac function in MI mice^230^. The different regulatory effects of exercise on TGFβ-Smad2/3 signaling pathway reflect the complexity of the anti-inflammatory mechanism of exercise.

Exercise may also improve cardiac dysfunction in HF rats by inhibiting the expression of lncRNA MALAT1, and the potential mechanism may be mediated by reducing cardiomyocyte apoptosis and increasing autophagy by regulating the miR-150-5p/PI3K/Akt signaling pathway^231^. Exercise also protects the heart from pressure overload-induced cardiac hypertrophy and inflammation by up-regulating miR-574-3p and inhibiting IL-6/JAK/STAT pathway^232^. Exercise-induced cardiac hypertrophy preconditioning can reverse TAC-induced cardiac fibrosis in HF mice and reduce apoptosis and oxidative stress by activating NRF2 and downstream signal transduction genes, suggesting that exercise-induced cardiac hypertrophy preconditioning may be a promising strategy for the prevention and treatment of cardiac fibrosis^233^.

In summary, the underlying mechanism of exercise intervention on inflammation in HF is complex, involving multiple molecules, transcription factors and non-coding RNAs, and even different regulatory effects on the same signaling pathway. So, how does exercise regulate the inflammatory response in HF through complex anti-inflammatory mechanisms? Our understanding is still limited.

## 6. Inflammation and exercise in other different cardiac diseases

HF is the end stage of various cardiac diseases. Inflammation is a common pathological feature across different cardiac diseases, including myocardial infarction, coronary artery disease, diabetic cardiomyopathy and hypertensive heart disease. Although the underlying mechanisms of inflammation may vary among those conditions, the role of exercise in modulating inflammatory responses and improving cardiac function is increasingly recognized. We will discuss the differences in inflammatory pathways and the effects of exercise intervention in various cardiac diseases in this section.

### 6.1 Myocardial infarction

Myocardial injury triggers the infiltration of neutrophils and monocytes/macrophages, with M1 macrophages predominating in the early stages. Those cells drive inflammatory responses by releasing pro-inflammatory factors such as IL-1β and TNF-α, while M2 macrophages participate in anti-inflammatory processes and tissue repair through IL-10 secretion^105^. Immune cells exacerbate oxidative stress and fibrosis by activating pathways including TGF-β/Smad, TLR4/MyD88/NF-κB, and NLRP3/caspase-1, leading to cardiomyocyte apoptosis and cardiac remodeling^234^, ^235^. Conversely, the activation of PI3K/Akt and Notch pathways promotes angiogenesis and cell survival^236^, ^237^. Exercise interventions (e.g., early moderate exercise or resistance training) mitigate the pathological processes by activating the SESN2/AMPK/PGC-1α pathway to suppress TGFB1 signaling and NLRP3 inflammasome activity, while shifting macrophage polarization from M1 to M2 phenotypes^238^, ^239^. This reduces pro-inflammatory cytokines (IL-1β, TNF-α), elevates anti-inflammatory IL-10 levels, decreases oxidative stress markers (e.g., MDA), and enhances antioxidant capacity (e.g., SOD). Furthermore, exercise promotes angiogenesis and proliferation of cardiac interstitial cells (e.g., telocytes), inhibits fibrotic markers (e.g., collagen deposition) and apoptosis-related proteins (Bax, Caspase-3), thereby improving cardiac function and attenuating tissue damage^239^. Modulation of miRNA-mRNA networks (e.g., upregulating miR-223-3p) further suppresses inflammatory signaling, ultimately achieving multi-target synergy to reduce adverse ventricular remodeling and promote myocardial repair^238^.

### 6.2 Coronary artery disease

The core inflammatory mechanism of coronary artery disease begins with lipid deposition in atherosclerotic plaques. Monocytes differentiate into macrophages and uptake oxidized low-density lipoprotein (ox-LDL) via scavenger receptors (CD36, SR-A), forming foam cells that release IL-6, TNF-α, and CRP^240^. Concurrently, Th1 cells secrete IFN-γ, promoting vascular smooth muscle cell apoptosis and MMPs expression, collectively exacerbating plaque instability^241^. Ox-LDL and inflammatory cytokines activate the NF-κB, JAK/STAT3, and MAPK pathways, driving endothelial adhesion molecule (VCAM-1, ICAM-1) expression, while ROS promote IL-1β and IL-18 maturation via the NLRP3 inflammasome, amplifying the inflammatory cascade^242^. Exercise intervention upregulates meteorin-like protein (Metrnl) secreted by skeletal muscle, inhibiting ROS-NLRP3 pathway activity, reducing IL-1β and TNF-α release, and improving endothelial aerobic metabolism while lowering oxidative stress, thereby delaying atherosclerosis progression^243^. Moderate-intensity continuous training significantly reduces CRP, fibrinogen, and von Willebrand factor (vWF) levels^244^. However, excessive exercise may exacerbate inflammation by increasing the coronary perivascular fat attenuation index (RCA-FAI), suggesting a U-shaped relationship between exercise intensity and anti-inflammatory effects^245^. Thus, personalized exercise regimens are essential to balance plaque stabilization and inflammation control.

### 6.3 Diabetic cardiomyopathy

The inflammatory mechanisms of diabetic cardiomyopathy are driven by chronic hyperglycemia and insulin resistance^246^. Advanced glycation end products (AGEs) activate the NF-κB pathway via the RAGE receptor, leading to the release of TNF-α, IL-6, and IL-1β, while excessive mitochondrial ROS generation further activates the NLRP3 inflammasome, exacerbating myocardial fibrosis and apoptosis^247^. Hyperglycemia-induced endoplasmic reticulum stress disrupts cardiomyocyte autophagy through the PERK/CHOP pathway, and the angiotensin II (Ang II)-activated RAAS system promotes pro-inflammatory macrophage polarization and TGF-β/Smad signaling, accelerating collagen deposition^248^. Insulin resistance-related lipid metabolism disorders trigger inflammation via the TLR4/MyD88 pathway due to elevated free fatty acids. Regular aerobic exercise improves insulin sensitivity by enhancing skeletal muscle glucose uptake, reducing AGEs accumulation and ROS production, while activating the AMPK/SIRT1 pathway to suppress NF-κB and NLRP3 inflammasome activity^249^. This upregulates IL-10 and adiponectin levels, reverses M1 macrophages polarization, and promotes M2 macrophages switching. Additionally, exercise regulates mitochondrial biogenesis (e.g., PGC-1α, TFAM) through FGF21 and Irisin, inhibits Drp1-mediated mitochondrial fission and TGF-β/Smad3 signaling, and significantly reduces myocardial IL-1β and Galectin-3 levels, thereby improving ventricular diastolic function^250^. Those mechanisms synergistically alleviate inflammation, oxidative stress, and fibrosis, offering novel non-pharmacological strategies for diabetic cardiomyopathy. Future research should focus on optimizing exercise protocols and identifying individualized biomarkers to enhance therapeutic precision.

### 6.4 Hypertensive heart disease

The inflammatory response in hypertensive heart disease is characterized by infiltration of monocytes/macrophages, CD8^+^ T cells, and Th17 cells, accompanied by reduced regulatory Tregs, leading to elevated pro-inflammatory cytokines (IL-17, IFN-γ, TNF-α, IL-1β) and decreased anti-inflammatory IL-10^251^. Immune cells drive inflammation and IL-1β release through activation of the PI3K/AKT/NF-κB pathway and NLRP3 inflammasome, while the MAPK and Rho/ROCK pathways promote vascular smooth muscle proliferation and oxidative stress^121^, ^251^. The TGF-β/Smad pathway mediates cardiac fibrotic remodeling. Exercise exerts multi-target protective effects by inhibiting NLRP3 pathway activity to reduce pro-inflammatory cytokine production^252^. Additionally, exercise improves endothelial NO bioavailability, reduces vascular oxidative stress and stiffness, and modulates the IL-6/IL-10 ratio while suppressing sympathetic overactivation and perivascular adipose tissue-derived inflammatory factor secretion^220^. Those integrated mechanisms collectively ameliorate cardiac metabolic microenvironments, reverse myocardial hypertrophy and fibrosis, and mitigate hypertension-associated cardiac inflammation and structural remodeling.

## 7. Conclusion and prospects

HF is a complex disease involving multiple biological pathways and cell types, and its treatment strategy requires a comprehensive consideration of multiple aspects of pathophysiology^4^. This article reviews the central role of inflammation in HF, including how the inflammatory response changes from an adaptive protective mechanism after myocardial injury to a chronic pathological process leading to cardiac dysfunction. We discuss in detail the dynamic changes of immune cells such as macrophages, neutrophils, T cells, B cells, etc. in the development of HF and how they affect cardiac structure and function by secreting cytokines and chemokines. In addition, the important role of non-immune cells such as cardiomyocytes, endothelial cells, fibroblasts, and extracellular matrix in HF and how they respond to inflammatory signals and participate in cardiac remodeling are explored.

Furthermore, the detailed understanding of the roles of various inflammatory cells and signaling pathways in HF pathogenesis reveals promising therapeutic targets. For instance, macrophages exhibit dynamic changes during HF progression, with pro-inflammatory macrophages contributing to cardiac dysfunction. Targeting macrophage polarization or selectively modulating macrophage subtypes could potentially mitigate pathological cardiac remodeling. Similarly, neutrophils, while essential for initial immune responses, can promote adverse remodeling when persistently activated. Strategies to regulate neutrophil activity or neutrophil extracellular trap (NET) formation could offer therapeutic benefits. T cells and B cells also play significant roles in HF progression, and modulating their activity or interactions could help in controlling inflammation and improving cardiac function. Moreover, targeting specific inflammatory signaling pathways such as TLR/MyD88/NF-κB, NLRP3 inflammasome, PI3K/Akt, and MAPK could provide novel therapeutic approaches to attenuate inflammation and cardiac remodeling.

Exercise intervention as part of HF treatment has been shown to be able to improve exercise endurance and quality of life in patients, and may have a positive impact on the inflammatory state^7^. This article summarizes how exercise can reduce the symptoms and progression of HF by modulating inflammatory mediators in the heart and circulation. Although exercise has been shown to reduce the levels of inflammatory markers and improve cardiac function, the exact mechanism of exercise intervention, especially at the molecular and cellular levels, still needs to be further elucidated. Future research should focus on elucidating the precise mechanisms by which exercise and other interventions modulate those inflammatory cells and pathways in HF. Additionally, developing and validating inflammatory biomarkers that can predict treatment response and prognosis may inform personalized therapeutic strategies.

In conclusion, inflammation is a key driver of HF progression, and targeting inflammatory cells and pathways holds significant therapeutic potential. Future studies are needed to further explore the intricate mechanisms underlying those processes and to develop novel, effective treatments that can improve the clinical outcomes and quality of life for HF patients.
